# Soy and Arabidopsis receptor-like kinases respond to polysaccharide signals from *Spodoptera* species and mediate herbivore resistance

**DOI:** 10.1038/s42003-020-0959-4

**Published:** 2020-05-08

**Authors:** Takuya Uemura, Masakazu Hachisu, Yoshitake Desaki, Ayaka Ito, Ryosuke Hoshino, Yuka Sano, Akira Nozawa, Kadis Mujiono, Ivan Galis, Ayako Yoshida, Keiichirou Nemoto, Shigetoshi Miura, Makoto Nishiyama, Chiharu Nishiyama, Shigeomi Horito, Tatsuya Sawasaki, Gen-ichiro Arimura

**Affiliations:** 1 Department of Biological Science and Technology, Faculty of Industrial Science and Technology, Tokyo University of Science, Tokyo, Japan; 2 Proteo-Science Center, Ehime University, Matsuyama, Japan; 3 Institute of Plant Science and Resources (IPSR), Okayama University, Kurashiki, Japan; 4 Faculty of Agriculture, Mulawarman University, Samarinda, Indonesia; 5 Biotechnology Research Center, The University of Tokyo, Tokyo, Japan; 6 Iwate Biotechnology Research Center, Kitakami, Iwate Japan; 7 Collaborative Research Institute for Innovative Microbiology, The University of Tokyo, Tokyo, Japan

**Keywords:** Molecular biology, Plant sciences

## Abstract

Plants respond to herbivory by perceiving herbivore danger signal(s) (HDS(s)), including “elicitors”, that are present in herbivores’ oral secretions (OS) and act to induce defense responses. However, little is known about HDS-specific molecules and intracellular signaling. Here we explored soybean receptor-like kinases (RLKs) as candidates that might mediate HDS-associated RLKs’ (HAKs’) actions in leaves in response to OS extracted from larvae of a generalist herbivore, *Spodoptera litura*. Fractionation of OS yielded Frα, which consisted of polysaccharides. The GmHAKs composed of their respective homomultimers scarcely interacted with Frα. Moreover, Arabidopsis HAK1 homomultimers interacted with cytoplasmic signaling molecule PBL27, resulting in herbivory resistance, in an ethylene-dependent manner. Altogether, our findings suggest that HAKs are herbivore-specific RLKs mediating HDS-transmitting, intracellular signaling through interaction with PBL27 and the subsequent ethylene signaling for plant defense responses in host plants.

## Abstract (web summary)

Uemura et al. study the mechanism of herbivore resistance in soybean and Arabidopsis. They show that receptor-like kinases (HAK1/2) respond to a polysaccharide in the oral secretions of *Spodoptera litura* and then interact with PBL27, resulting in an ethylene-dependent herbivore resistance.

## Introduction

To defend themselves against pest attack, plants are equipped with sophisticated sensory systems to perceive herbivore-derived danger signals (HDSs), including “elicitors”, that induce defense responses^1^. Herbivore oral secretions (OS) of several lepidopteran larvae and other arthropods contain an array of elicitors, including hydroxy fatty acid-amino acid conjugates (FACs; e.g., volicitin [*N*-(17-hydroxylinolenoyl)-l-glutamine])^2–4^. Although it has been shown that FAC-type elicitors induce depolarization of the plant cell plasma-membrane potential by activating voltage-dependent Ca^2+^ channels^5^, specific receptors for FACs have not been characterized^6^. Other distinct types of HDSs have been characterized from several herbivore species, such as β-glucosidase from cabbage white butterfly (*Pieris brassicae*)^7^, disulfooxy fatty acids (caeliferins) from the American bird grasshopper (*Schistocerca americana*)^8^, the peptide inceptins from fall armyworm (*Spodoptera frugiperda*)^9^, the peptide tetranins from spider mites (*Tetranychus urticae*)^10^, a porin-like protein from Egyptian cotton leafworm (*Spodoptera littoralis*)^11^, and a putative β-galactofuranose polysaccharide from *S. littoralis*^12^.

There is very little knowledge about the HDS recognition system and its downstream intracellular signaling in plants. One notable report showed that rice leucine-rich repeat (LRR) receptor-like kinase 1 (OsLRR-RLK1) is involved in defense responses of rice against striped stem borer (SSB)^13^. In other cases, rice and tobacco lectin receptor kinases (LecRKs) were shown to serve positively and negatively for defense responses of the host plants during damage by the herbivores *Nilaparvata lugens* and *Manduca sexta*, respectively^14,15^. Moreover, it has been shown that the Arabidopsis LRR-RLK, brassinosteroid insensitive1-associated kinase 1 (BAK1), is required for the defense response to aphid attack^16^. However, it remains to be clarified, for all of these membrane proteins, whether these proteins are specific to elicitors from the respective insects, and if so, what type of elicitor molecule(s) act as the HDS. More recently, a cowpea LRR-receptor-like protein (RLP) was shown to serve as inceptin receptor (INR), which is responsible for elicitor-induced responses and enhanced defense against armyworms (*Spodoptera exigua*) (https://www.biorxiv.org/content/10.1101/679803v1).

In contrast to the scant knowledge about HDSs, microbe-associated molecular patterns (MAMPs), including saccharide elicitors (e.g., chitin) and proteinaceous elicitors (e.g., flagellin and EF-Tu) have been well characterized, and found to be recognized by pattern recognition receptors (PRRs) located at the plasma membrane, including RLKs and RLPs^17,18^. For instance, chitin elicitor receptor kinase 1 (CERK1) is a member of an RLK family whose members are broadly involved in recognizing pathogenic saccharide elicitors (e.g., chitin, peptidoglycan, lipopolysaccharide, and β-glucan)^19^. In turn, BAK1, one of the LRR-RLKs that is bound to other LRR-RLK/RLP members, plays a crucial role in the recognition of proteinaceous elicitors (e.g., flagellin, EF-Tu, SCFE1, NLP, and endogenous Atpep)^18^. Following the perception of these MAMPs, CERK1-LYK5, a chitin receptor complex, transmits phosphorylation signals to receptor-like cytoplasmic kinase AtPBL27 and RLCK VII-4 members to activate the MAPK cascade for defense response in Arabidopsis^20–23^. Likewise, in addition to these aboveground systems, in rhizobial, symbiotic and immune signaling of soybeans, lipo-chitooligosaccharide Nod factors from rhizobial symbionts appear to be recognized via GmNFR1/GmNFR5 (lysine-motif (LysM)-RLK)^24^. Thus, PRRs (RLKs/RLPs) might be involved in a broad range of interactions between host plants and their partners or competitors, but to date, only a few HDS-specific RLKs have been characterized.

In the current study, we focused on soybean RLKs that act for recognition of elicitor(s) that are present in the OS of the generalist herbivore *Spodoptera litura*, and for the subsequent intracellular immune signaling. Here we show that soybean plasma membrane-localized HDS-associated receptor-like kinases (HAKs) play a significant role in eliciting defense responses of host plants mediated through an array of intracellular signaling activations.

## Results

### Mining of the soybean HAKs

First, we focused on soybean genes that are homologs to Arabidopsis CERK1 (AtCERK1), which is involved in recognizing an array of pathogen-derived oligosaccharide elicitors, as described above^19^. Given that high molecular weight substances consisting of unknown oligosaccarides in OS of *Spodoptera* spp. function as putative elicitors in Arabidopsis host plants^12^, we focused on the RLK superfamily based on our hypothesis that some soybean HAKs (GmHAKs) are structurally similar to AtCERK1 (LysM-RLK), which may recognize various unknown elicitors. To identify and obtain the full-length open reading frame (ORF) sequence of putative soybean RLK cDNA clones based on in silico analyses of sequence similarity to AtCERK1, we then performed a BLASTX search using the ORF sequence of AtCERK1 using a Legume Base NBRP (https://www.legumebase.brc.miyazaki-u.ac.jp/) supported by the NBRP project (*Lotus japonicus* and *Glycine max*) office, which provides resources of full-length cDNA clones publically. Since the BLASTX search hit only a single clone of LysM-RLK (GMFL01-15-B07), we also expanded the scope of our interest in potential candidates to other structural variants of RLKs (LRR-RLKs and proline-rich extensin-like receptor kinases [PERKs]; Supplementary Table 1). As a result, we mined 19 soybean RLKs showing amino acid sequence similarity to AtCERK1 (~44% identities). Of these 19 putative soybean RLKs thus far mined as GmHAK candidates, the corresponding cDNAs of 15 genes were successfully amplified (Supplementary Fig. 1a), and we then generated the respective 15 transgenic Arabidopsis lines independently expressing RLK genes under the control of the constitutive cauliflower mosaic virus (CaMV) 35S promoter (35SP) (R1-R15) (Supplementary Table 1).

**Fig. 1:**
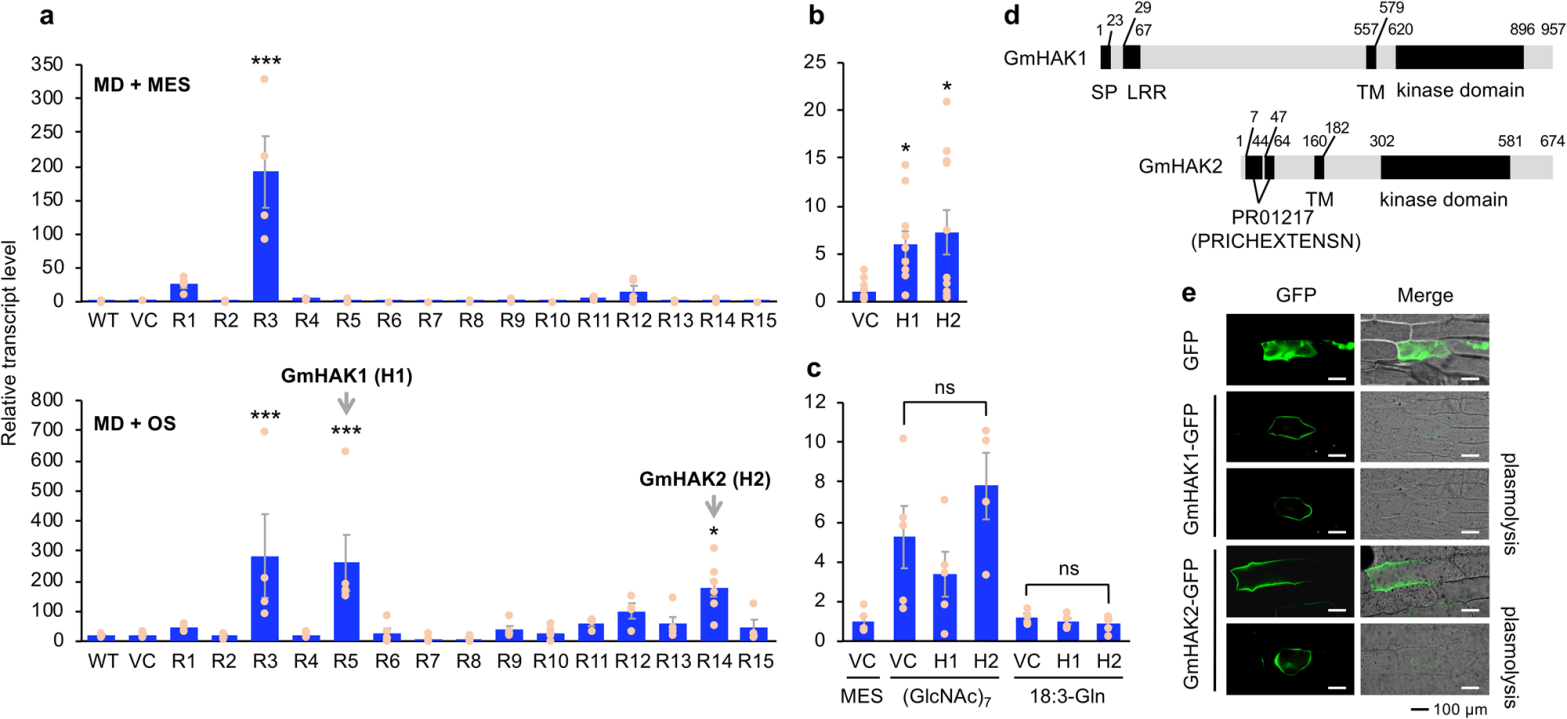
Mining of GmHAKs and their structure and subcellular localization. Transcript levels of *PDF1.2* in leaves of wild-type (WT) plants, vector control (VC) plants, and 15 lines of GmHAK-expressing Arabidopsis plants (R1-R15) at 24 h after mechanical damage (MD) with application of MES buffer or OS from *Spodoptera litura* (**a**, *n* = 4–8). Moreover, VC, H1 (R5), and H2 (R14) lines were damaged with *S. litura* larvae (**b**, *n* = 10–11) or treated with MD + MES buffer, chitin oligosaccharides [(GlcNAc)_7_], or *N*-linolenoyl-l-Gln (18:3-Gln) (**c**, *n* = 4–5) for 24 h, and transcript levels of *PDF1.2* in their leaves were measured. All the individual data points are shown with the means and standard errors. Data marked with an asterisk are significantly different from those of WT (**a**) or VC (**b**), based on an ANOVA with Holm’s sequential Bonferroni post hoc test (***P < 0.001; *0.01 ≤ *P* < 0.05). ns, not significant. **d** GmHAK1 and GmHAK2 proteins are schematically represented. LRR, leucine-rich repeat; SP, signal peptide; PR01217 (PRICHEXTENSN), proline-rich sequence; TM, transmembrane domain. **e** Subcellular localization of GmHAKs. The vector containing the CaMV 35S promoter (35SP)::GFP or 35SP:: GmHAK1 or GmHAK2 fused to GFP (GmHAK1-GFP or GmHAK2-GFP, respectively) was transformed into the epidermal cells of onions. Plasmolysis was induced by 0.6 M mannitol for 15 min. The photograph with GFP signal alone and the photograph with merged GFP signal and bright field images are shown.

The defense ability of the transgenic lines was evaluated by monitoring the transcript levels of the jasmonate (JA)- and ethylene-inducible defensin gene *PDF1.2*^25^ in leaves subjected to mechanical damage (MD) alone, or to MD with exogenous application of OS collected from *S. litura* larvae. Both MD alone and MD + OS enhanced *PDF1.2* transcript levels in leaves of the R3 line in comparison to the levels in wild-type (WT) leaves (Fig. 1a). Note that the R3 protein has a GUB_WAK_bind domain, which is conserved in wall-associated kinases (WAKs), the specific receptors for oligogalacturonides released from the plant cell wall^26^, implying that R3-RLK has the same or similar function as Arabidopsis WAKs. In contrast, the R5 and R14 lines showed elevated *PDF1.2* transcript levels only with MD + OS, relative to the levels in WT. These responses were confirmed using other genotypes of the identical foreign genes (R5-2 and R14-2), but not MD-inducible *trypsin inhibitor* (*TI*) expression (Supplementary Fig. 2), which further confirmed that R5 (H1)- and R14 (H2)-corresponding genes were responsible for defense responses in an OS-specific manner. We therefore redesignated the putative receptors expressed in transgenic lines R5 and R14 as GmHAK1 and GmHAK2, respectively, and the corresponding transgenic lines were renamed H1 and H2, and are so referred to hereafter.

### GmHAKs are specific RLK candidates for *Spodoptera* OS

H1 and H2 leaves responded dramatically to not only *S. litura* OS but also to damage by *S. litura* by expressing *PDF1.2*, in comparison to those of the vector control (VC) plants (Figs. 1a, b). However, H1 and H2 leaves did not respond to MD plus other types of elicitors, including chitin oligosaccharides [(GlcNAc)_7_] and *N*-linolenoyl-l-Gln (18:3-Gln, a FAC elicitor from several lepidopteran larvae^4^), as indicated by their expression of *PDF1.2* at similar levels to those in the vector control (VC) plants (Fig. 1c). Moreover, *PDF1.2* transcript levels were not induced in Arabidopsis leaves by application of OS collected from the larvae of other lepidopteran species (*Mythimna loreyi* and *Pieris rapae*) (Supplementary Fig. 3a). Altogether, these findings imply that GmHAK1 and GmHAK2 are not likely to be responsive to the classical elicitors chitin and FAC (another elicitor that is present at very high levels in *S. litura* OS).

### Structure and plasma-membrane localization of GmHAKs

Plant RLKs have a typical structure including a ligand-binding domain in the extracellular region, a single transmembrane region and a kinase domain in the intracellular region, and are located at the plasma membrane, where they function in perception of external stimuli such as elicitors^18^. Notable characteristics of GmHAK proteins were that they each possess a single predicted transmembrane region and a kinase domain (Fig. 1d), and that a leucine-rich repeat (LRR) and a proline-rich sequence (PR01217 [PRICHEXTENSN]) are present in the extracellular region of GmHAK1 and GmHAK2, respectively. Examining the subcellular localization of the GmHAKs by transient expression of GmHAK-GFP (green fluorescent protein) fusion protein in the epidermal cells of onion showed GFP signals of GmHAK1 and GmHAK2 along the plasma membrane (Fig. 1e).

### Fractionation and characterization of OS components

**Fig. 2:**
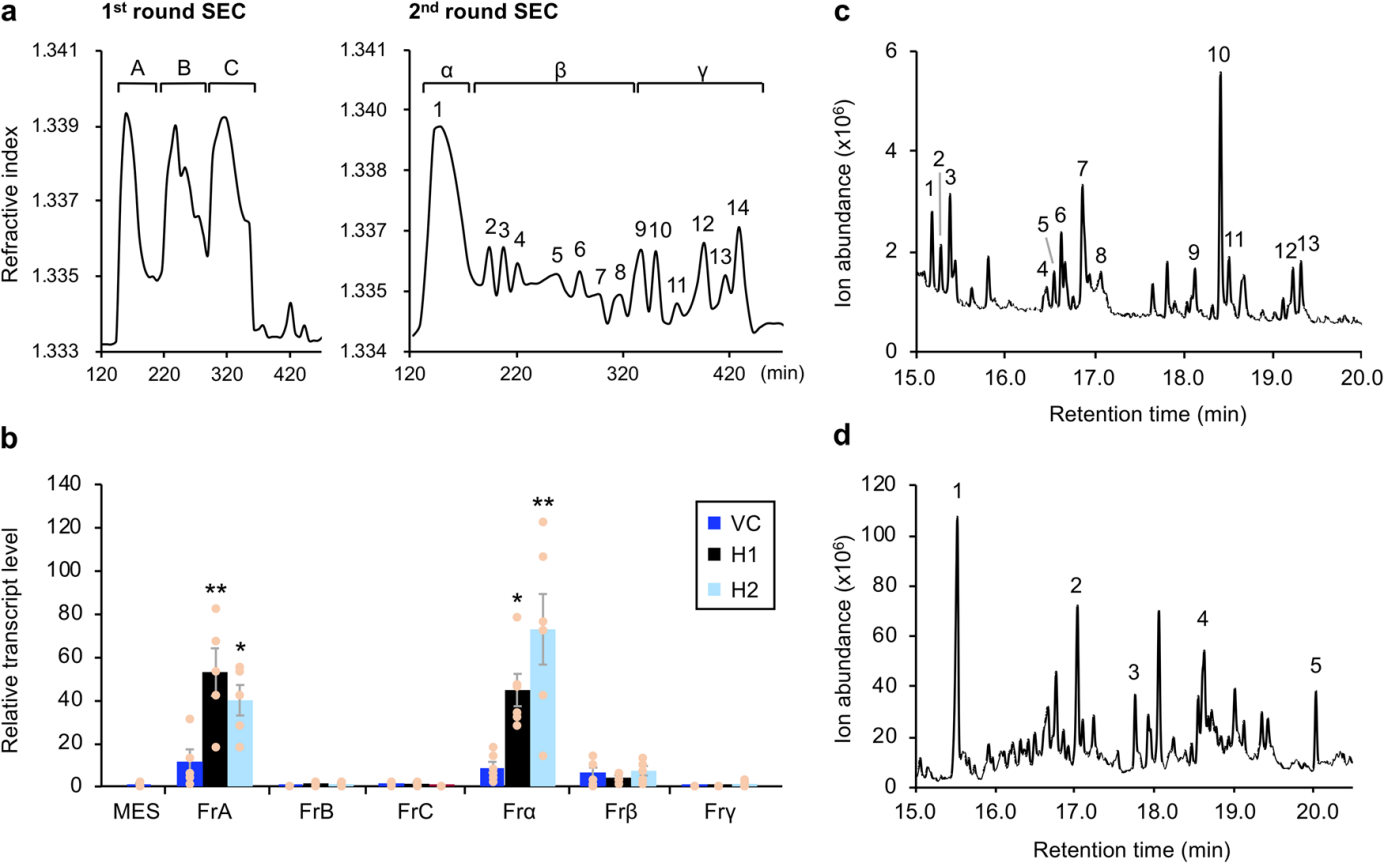
Fractionation and identification of GmHAK-influenced components in *Spodoptera litura* OS. **a** The first round of size exclusion chromatography (SEC) of *S. litura* OS showed three major fractions (FrA, FrB, and FrC) ranging in molecular size from 100 to 1800 Da (left). FrA was subjected to the second-round SEC ranging in molecular size from 1500 to 20,000 Da, resulting in 14 peaks that were grouped into three fractions (Frα, Frβ, and Frγ) (right). **b** Transcript level of *PDF1.2* in leaves of vector control (VC) and GmHAK-expressing lines (H1 and H2) of Arabidopsis plants at 24 h after mechanical damage (MD) with application of MES buffer or each OS fraction. All the individual data points are shown with the means and standard errors (*n* = 4–6). Data marked with an asterisk are significantly different from those of VC, based on an ANOVA with Holm’s sequential Bonferroni post hoc test (**0.001 ≤ *P* < 0.01; *0.01 ≤ *P* < 0.05). **c** Total ion chromatogram of GC-MS for monosaccharide determination analysis of Frα. **d** Total ion chromatogram of GC-MS for monosaccharide branching points determination analysis of Frα. The peaks marked with numbers indicate the acetylated methyl glycoside derivatives. Their fragmentation patterns are shown in Supplementary Fig. 6.

Based on the experimental strategy employed in a previous study in which the OS from *S. littoralis* (closely related to *S. litura*) was fractionated^12^, we fractionated *S. litura* OS by size exclusion chromatography (SEC). Through the first round of SEC to separate components with molecular sizes of 100–1800 Da, three distinct groups consisting of major fractions (FrA, FrB, and FrC) were obtained (Fig. 2a). Of these, FrA was shown to induce elevated transcript levels of *PDF1.2* in mechanically damaged VC leaves and to induce more drastically increased levels in H1 and H2 leaves (Fig. 2b). Through the second round of SEC to separate FrA into fractions with molecular sizes of 1500–20,000 Da, 14 major peaks were detected (Fig. 2a). Of the three major fractions (Frα [which contained peak 1], Frβ [which consisted of peaks 2–7], and Frγ [which consisted of peaks 8–14]), Frα, containing the highest molecular weight components, was able to increase the transcript levels of *PDF1.2* in mechanically damaged leaves of the H1 and H2 lines to a higher level than those in VC leaves (Fig. 2b). Both FrA and Frα also induced the transcript levels of a *pathogenesis-related* (*PR*) gene in mechanically damaged soybean leaves (Supplementary Fig. 4).

Frα was not subjected to further fractionation by SEC because there was not an sufficient total quantity of material in it. We instead shifted to an alternative strategy to fractionate FrA, using normal-phase chromatography and reverse phase chromatography. Fractionation through reverse phase chromatography was not able to separate FrA into distinct components (Supplementary Fig. 5a). However, fractionation through normal-phase chromatography produced a major component peak, eluted with 40% water (FrII), which induced a higher level of the *PDF1.2* transcript in mechanically damaged leaves of the H1 and H2 lines with respect to VC plants (Supplementary Figs. 5a, b). Moreover, when FrA was heated at 95 °C for 20 min or incubated with a 1% (w/w) protease cocktail at 37 °C overnight, its ability to induce an increase of the *PDF1.2* transcript level was not impaired (Supplementary Fig. 3b), suggesting that the elicitor molecule(s) in the FrA subfractions Frα or FrII contained a single or multiple macromolecule(s) but did not mainly consist of protein(s) or peptide(s), in agreement with previous reports^12^.

^1^H-NMR analysis was performed to make a rough survey of the molecular species present in Frα and FrII (Supplementary Figs. 5c, d). We observed overlapping broad signals in the region of 3.2–4.5 ppm, which were typical of chemical shifts caused by sugar ring protons. These results were in accord with data from gas chromatography–mass spectrometry (GC-MS) analysis of acetylated methyl glycosides^27^, which showed fragmentation of the Frα components into hexoses (e.g., glucose, galactose and mannose) and a minor group of pentoses (e.g., arabinose, ribose and xylose) (Fig. 2c, Supplementary Fig. 6a). Furthermore, analysis of monosaccharide branching points^28^ of Frα showed the presence of pentofuranoside, hexopyranoside, 3-substituted hexopyranoside, and 3,6-di-substituted hexopyranoside. We therefore predicted that the FrA-fractionated Frα or FrII (predicted size >15 kDa) is a polysaccharide(s) containing deoxy sugar, or a complex macromolecule composed of sugar (Fig. 2d, Supplementary Fig. 6b).

Finally, the possibility that the artificial diet consumed by larvae might contribute to the metabolites and elicitor activity of OS was considered. To examine this possibility, we assessed the OS fractions collected from *S. litura* larvae that had been reared on Arabidopsis or soybean host plants. The OS fractionation resulted in distinct chromatographs with components ranging in size from 100 to 1800 Da (Supplementary Fig. 7a), but FrA (containing the highest molecular weight components) was most active in inducing the transcript level of *PDF1.2* in mechanically damaged leaves of WT in both cases (Supplementary Fig. 7b). FrA induced stronger responses in the H1 and H2 lines than in the VC line (Supplementary Fig. 7c).

### Homomultimers of GmHAKs do not bind Frα

**Fig. 3:**
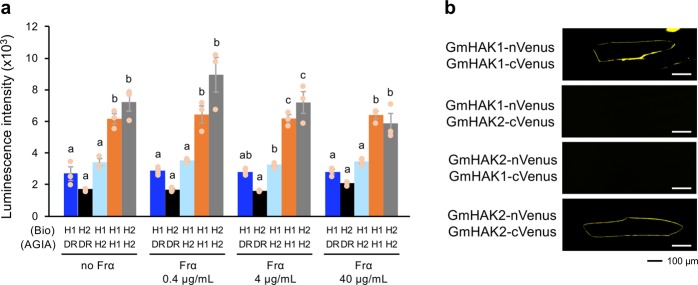
Molecular interactions of GmHAKs. **a** Luminescence intensities based on the AlphaScreen assay to assess the interactions between AGIA-conjugated proteins (AGIA) and biotinylated-proteins (Bio) in the presence and absence of Frα. All the individual data points are shown with the means and standard errors (*n* = 3). Means indicated by different small letters are significantly different, based on an ANOVA with post hoc Tukey’s HSD (*P* < 0.05). Recombinant proteins synthesized using a cell-free system are presented in Supplementary Fig. 12. DR, *Escherichia coli* dihydrofolate reductase serving as control; H1, GmHAK1; H2, GmHAK2. **b** Bimolecular fluorescence complementation (BiFC) analysis of GmHAK interactions. GmHAK1 or GmHAK2 fused to the N-terminal fragment of Venus (nVenus) and GmHAK1 or GmHAK2 fused to the C-terminal fragment of Venus (cVenus) were co-expressed in the epidermal cells of onions.

To investigate the molecular interaction between HAKs and elicitors, we performed isothermal titration calorimetry (ITC) analysis using Frα and the extracellular domain of GmHAKs. No change of heat capacity was observed by the titration of Frα to cells containing GmHAKs or GFP control protein (Supplementary Fig. 8). Interestingly, GmHAK1 and GmHAK2 were each able to form homodimers, and these interactions were not dependent on the presence of Frα, according to the results of assays using the AlphaScreen system (Fig. 3a). These results were in accord with the observation of the interactions in vivo using a bimolecular fluorescence complementation (BiFC) assay. For that assay, proteins consisting of GmHAK1 or GmHAK2 fused to the N-terminal portion of Venus (GmHAK1-nVenus or GmHAK2-nVenus) and the C-terminal portion of Venus (GmHAK1-cVenus or GmHAK2-cVenus) were transiently expressed in the epidermal cells of onions (Fig. 3b). The fluorescent signals of these recombinant Venus proteins were detected along the plasma membrane upon expression of GmHAK1-nVenus with GmHAK1-cVenus, or GmHAK2-nVenus with GmHAK2-cVenus. However, the signals were not detected upon expression of GmHAK1-nVenus with GmHAK2-cVenus, or GmHAK2-nVenus with GmHAK1-cVenus (Fig. 3b). All of these findings indicate that homomultimers of GmHAKs may serve as Frα-non-interacting or low-affinity Frα-binding RLKs.

### Function of GmHAKs in soybean defense responses

**Fig. 4:**
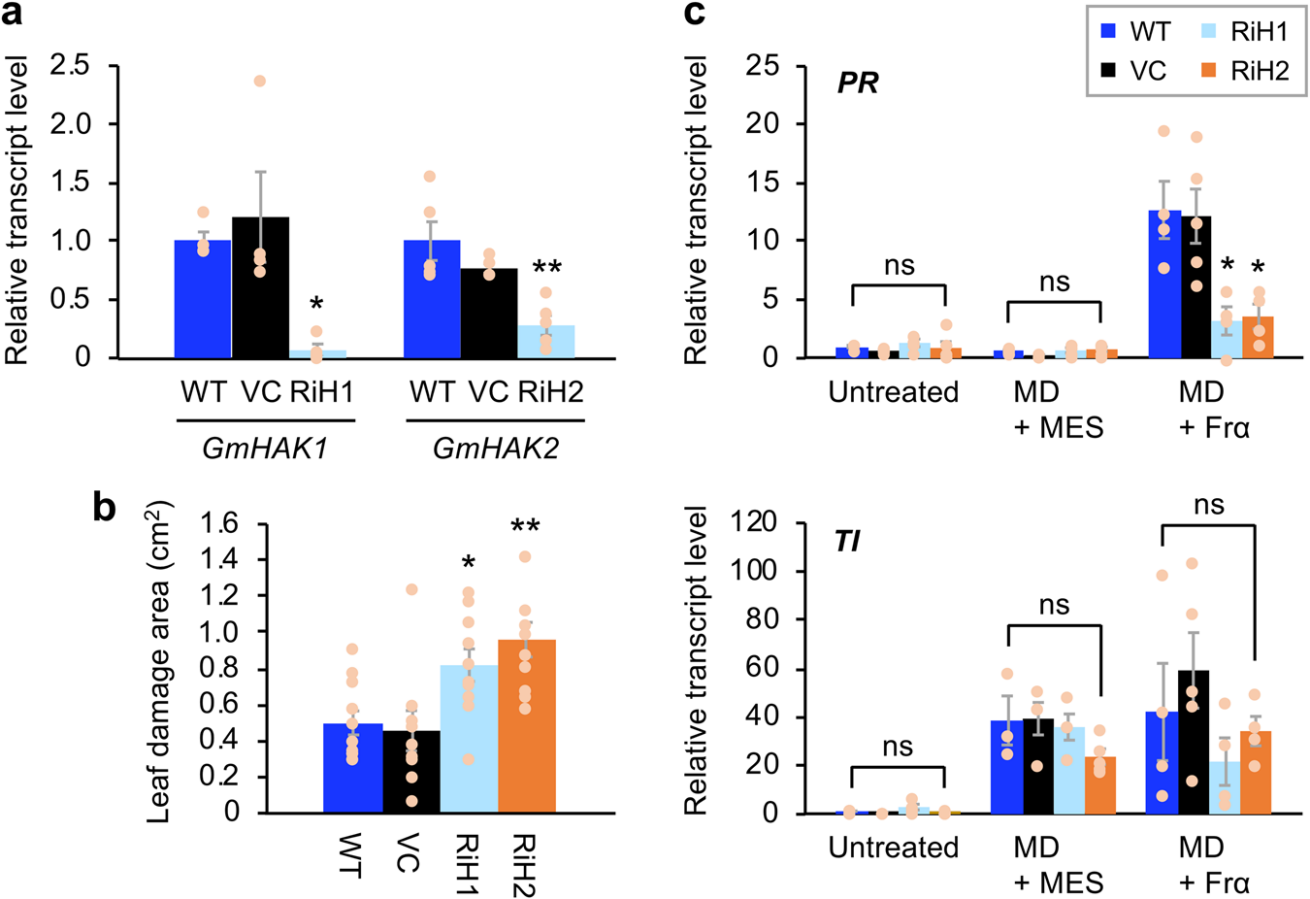
Defensive properties of GmHAK RNAi lines. **a** Transcript levels of *GmHAK1* or *GmHAK2* gene in leaves of wild-type (WT) plants, vector control (VC) plants, and RNAi lines of GmHAK1 (RiH1) and GmHAK2 (RiH2) (*n* = 4–5). **b** A *Spodoptera litura* larva was put onto the leaf of a potted plant. The area of the leaf damage after 24 h was determined (*n* = 9–11). Transcript levels of defense genes (*pathogenesis-related* [*PR*] and *trypsin inhibitor* [*TI*]) (**c**) in untreated leaves and the leaves at 24 h after mechanical damage (MD) with application of MES buffer or Frα (*n* = 3–5). All the individual data points are shown with the means and standard errors. Data marked with an asterisk are significantly different from those of WT, based on an ANOVA with Holm’s sequential Bonferroni post hoc test (**0.001 ≤ *P* < 0.01; *P < 0.05; ns, *P* > 0.05).

In order to assess the intrinsic function of GmHAKs in soybean defense responses, we obtained GmHAK knockdown transgenic soybean lines by employing the virus-induced gene silencing system using Apple latent spherical virus (ALSV) vectors^29^. In the soybean plants that had been inoculated with ALSV (lines RiH1 and RiH2), the levels of accumulation of *GmHAK1* and *GmHAK2* transcripts in leaves decreased to 6.6% and 27.9% in comparison to those in WT leaves, respectively (Fig. 4a). Instead, *GmHAK1* homolog (*GLYMA11G200300*, 57% amino acid sequence identity to *GmHAK1*; Supplementary Fig. 1b) or *GmHAK2* homolog (*GLYMA07G004700*, 57% amino acid sequence identity to *GmHAK2*; Supplementary Fig. 1b) did not show decreased transcript levels in leaves of RiH1 and RiH2, respectively, supporting the idea that knockdown of genes occurred specifically on the corresponding genes (Supplementary Fig. 9).

The knockdown plants exhibited more leaf damage by *S. litura* larvae compared with the damage exhibited by WT and VC plants (Fig. 4b). These results were in accord with the fact that the transcript levels of a *PR* gene in these knockdown plant leaves subjected to MD + Frα treatment were lower than those in WT and VC leaves (25.2% and 28.1%, respectively, of those in WT) (Fig. 4c). The transcript level of the gene for *TI*, a wound-responsive defense gene, was strongly induced by MD in all of the plant lines assessed, but MD + Frα treatment did not have significant effects on the transcript levels compared with MD alone (Fig. 4c).

### Arabidopsis HAK and early signaling components in Arabidopsis

**Fig. 5:**
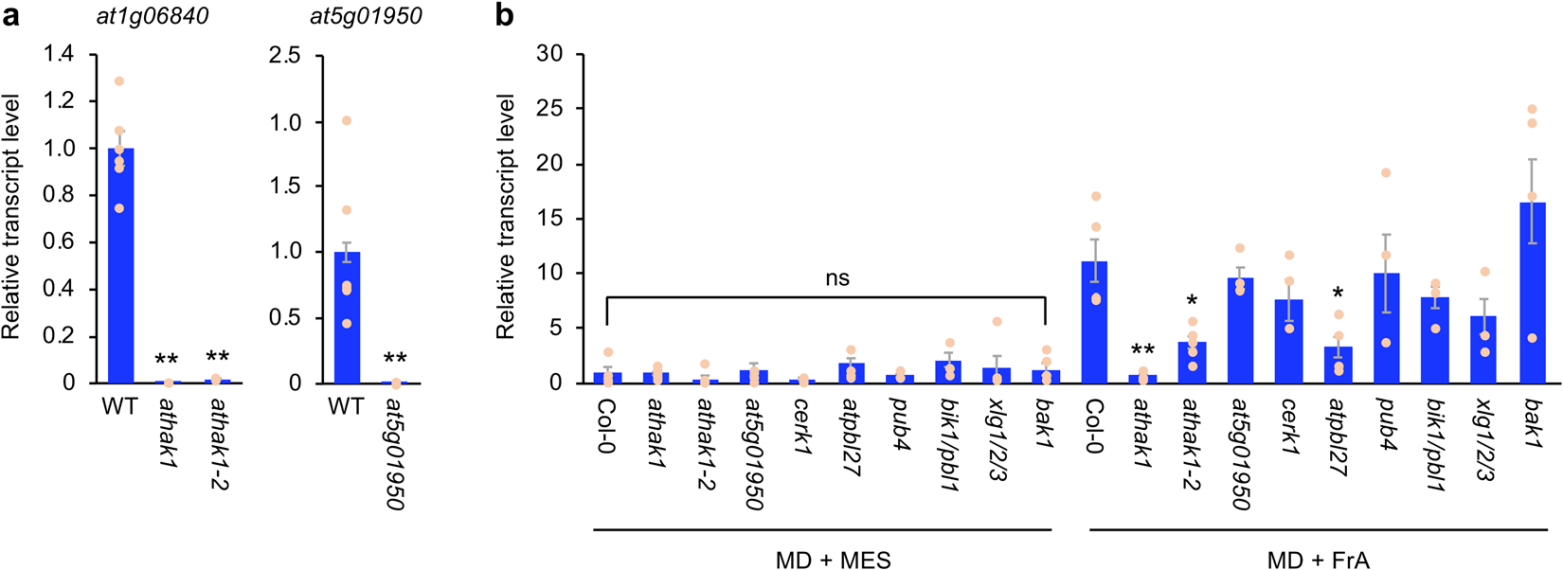
Mining of *GmHAK1*-homologous genes and intracellular signaling components involved in FrA-induced transcription of *PDF1.2*. **a** Transcript levels of GmHAK1-homologous genes (*at1g06840* [*AtHAK1*] and *at5g01950*) in leaves of the respective mutants and the wild-type (WT) (*n* = 4–6). **b** Transcript levels of *PDF1.2* in leaves of the respective mutants and the WT at 24 h after mechanical damage (MD) with application of MES buffer or FrA (*n* = 4–8). All the individual data points are shown with the means and standard errors. Data marked with an asterisk are significantly different from those of WT, based on an ANOVA with Holm’s sequential Bonferroni post hoc test (**0.001 ≤ *P* < 0.01; *0.01 ≤ *P* < 0.05; ns, *P* > 0.05).

**Fig. 6:**
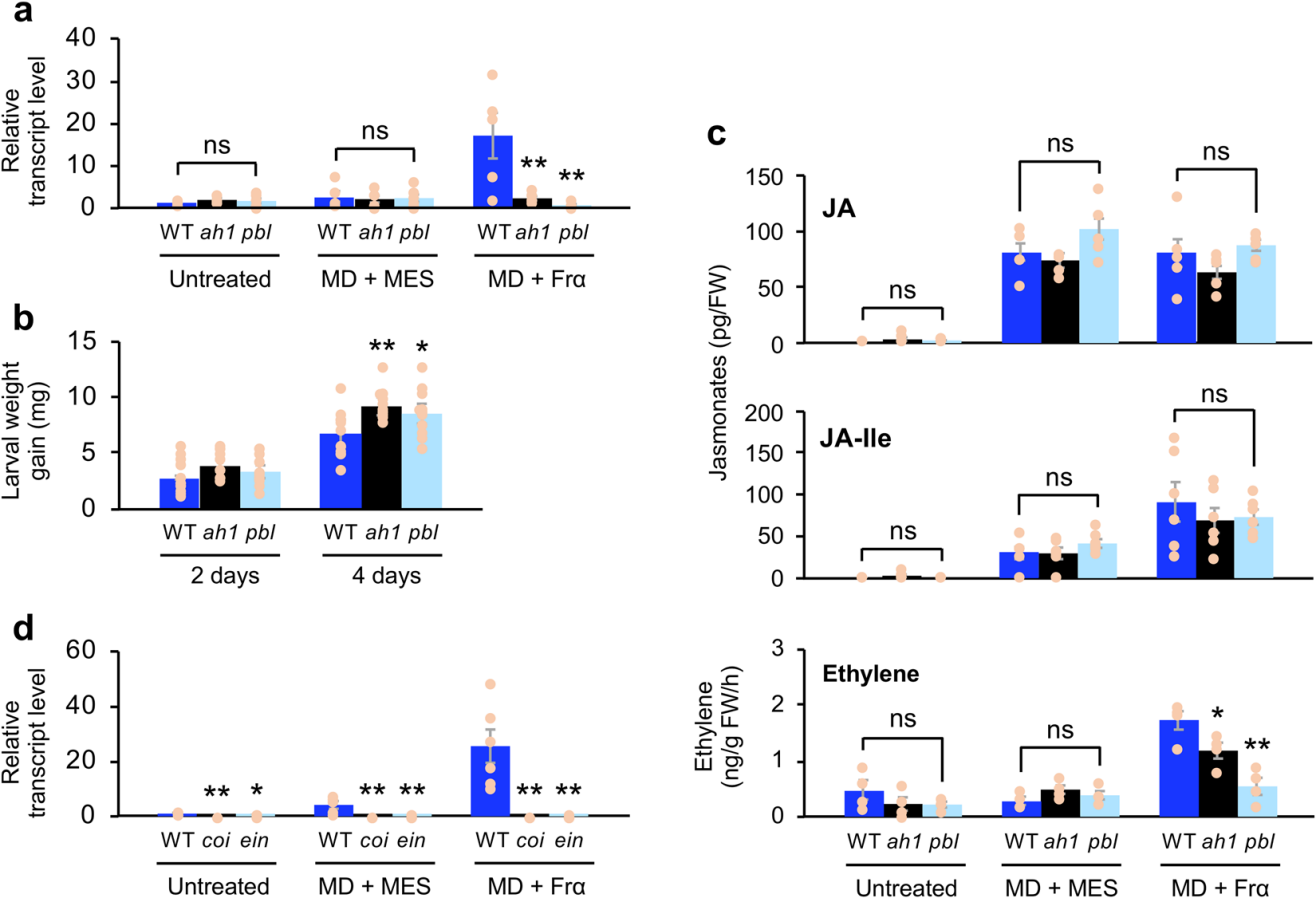
Physiological characterization of AtHAK1 and AtPBL27. Transcript levels of *PDF1.2* (**a**) and de novo accumulation levels of jasmonates (jasmonic acid (JA), jasmonoyl-l-isoleucine (JA-Ile)) and ethylene (**c**) in untreated leaves of Arabidopsis wild-type (WT), T-DNA insertion mutant of AtHAK1 (*athak1* [*ah1*]), and AtPBL27 (*atpbl27* [*pbl*]) plants and the leaves at 24 h after mechanical damage (MD) with application of MES buffer or Frα. **b** The net body weight that *Spodoptera litura* larvae gained during 2 and 4 days. **d** Transcript levels of *PDF1.2* in untreated leaves of WT, *coi1-1* (*coi*), and *ein2-1* (*ein*), and leaves at 24 h after MD with application of MES buffer or Frα. All the individual data points are shown with the means and standard errors (*n* = 4–6, 12, 4–6 and 4–6 for (**a**), (**b**), (**c**), and (**d**), respectively). Data marked with an asterisk are significantly different from those of WT, based on an ANOVA with Holm’s sequential Bonferroni post hoc test (**0.001 ≤ *P* < 0.01; *0.01 ≤ *P* < 0.05; ns, *P* > 0.05). FW, fresh weight.

In order to deepen our understanding of plant intracellular signaling after OS perception, we next explored Arabidopsis genes homologous to GmHAKs. Based on BLASTP searches using the extracellular region sequence of both GmHAKs, we mined two AtHAK candidate genes showing amino acid sequence similarity to GmHAK1 (at1g06840 [57% identity] and at5g01950 [59% identity]). Of them, one T-DNA insertion mutant line (*athak1*, corresponding to at1g06840; Fig. 5a) showed a lower expression level of *PDF1.2* in leaves, in comparison to that in WT leaves, after MD + FrA treatment (Fig. 5b, Supplementary Fig. 1b). This was confirmed by the similar response of another T-DNA insertion mutant line (*athak1-2*) to MD + FrA, as well as the response of *athak1* to MD + Frα (Figs. 5b, 6a). On the other hand, when response abilities of the other T-DNA insertion mutant lines, corresponding to AtPBL27, PUB4, BIK1/PBL1, and XLG1/2/3 (well characterized as MAMP-associated kinases, E3 ubiqutin ligase or G proteins that act via a recognition complex and intracellular signaling), to MD + FrA treatment were assessed, a weaker response of *PDF1.2* expression in AtPBL27 mutant (*atpbl27*) leaves was observed, in comparison to that in WT leaves (Fig. 5b). Again, as *atpbl27* showed a weaker response to MD + Frα (Fig. 6a), we confirmed that AtHAK1 (at1g06840) and AtPBL27 (at5g18610) significantly contributed to FrA/Frα responses promoting Arabidopsis defense properties. These conclusions were in agreement with the observation that *athak1* and *atpbl27* exhibited enhanced development of *S. litura* larvae on the potted plants during 4 days (Fig. 6b). On the other hand, in response to either MD alone or MD + Frα, the endogenous levels of jasmonates (jasmonic acid [JA] and jasmonoyl-l-isoleucine [JA-Ile]) did not differ among WT and mutant leaves (Fig. 6c). In contrast, both *athak1* and *atpbl27* mutant leaves showed lower emission of ethylene in response to MD + Frα, compared with that from WT leaves (Fig. 6c). Our present findings accorded with the much lower expression level of *PDF1.2* in leaves of *ein2-1* (ethylene insensitive mutant^30^) in response to MD + Frα, compared with that in WT leaves (Fig. 6d). However, the *PDF1.2* expression was also attenuated in the leaves of *coi1-1* (jasmonate insensitive mutant^31^) (Fig. 6d), indicating altogether that Frα committed Arabidopsis to both biosynthesis and signaling of ethylene, whereas it committed this plant to jasmonate signaling but not jasmonate biosynthesis.

Note that neither the *athak1* nor *atpbl27* mutant showed any marked differences in plant growth, development or morphology (Supplementary Fig. 10a). Moreover, those mutants did not show impaired responses to jasmonates or ethylene, as root and hypocotyl development of *athak1* or *atpbl27* seedlings were observed to occur similarly to those in WT seedlings in the presence of methyl jasmonate (MeJA) and an ethylene precursor, 1-aminocyclopropane-1-carboxylic acid (ACC), in contrast to the impairment of such development in *coi1-1* and *ein2-1* in the presence of MeJA and ACC, respectively (Supplementary Fig. 10b).

**Fig. 7:**
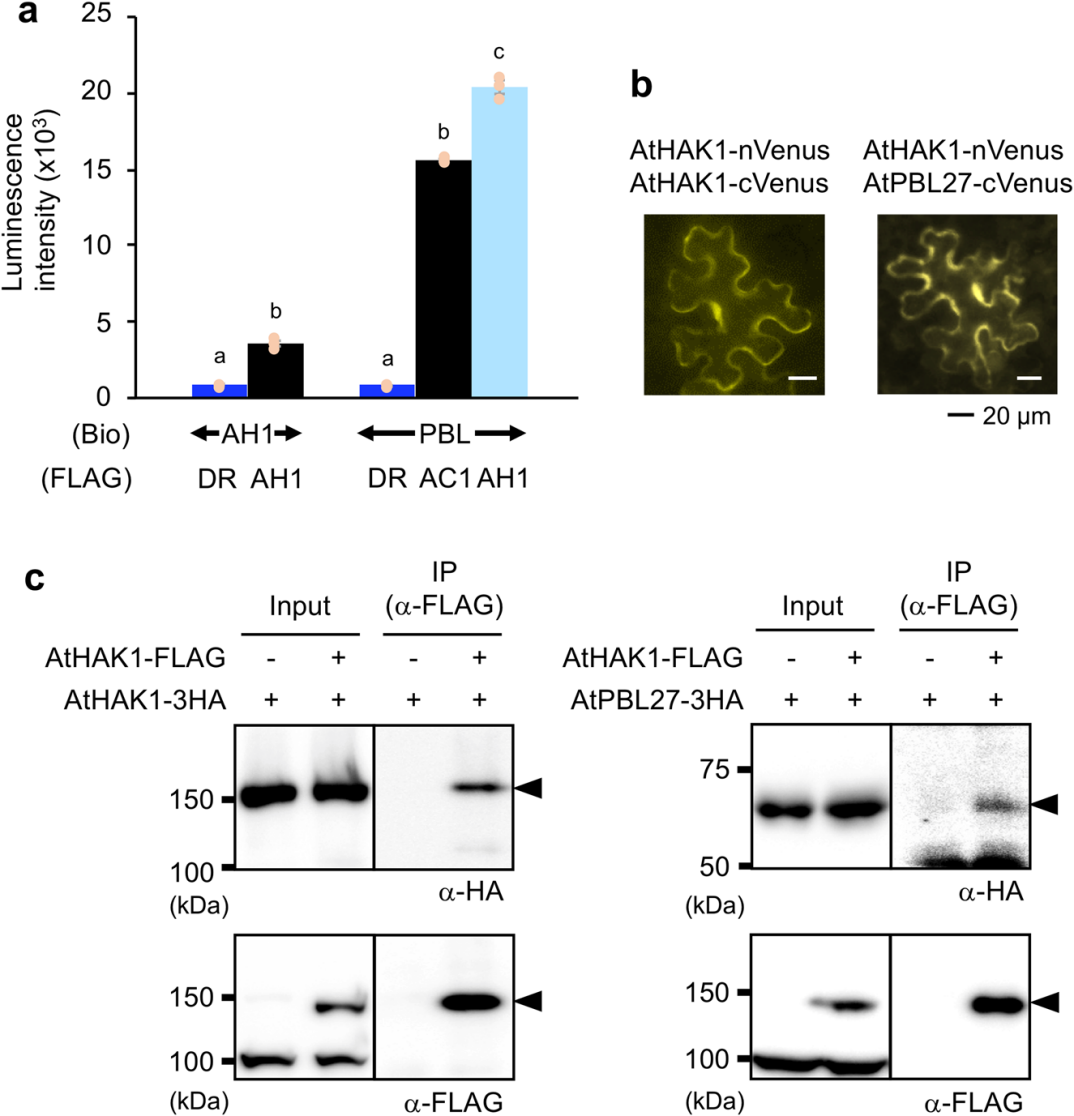
Interactions of AtHAK1 and AtPBL27. **a** Luminescence intensities based on the AlphaScreen assay to assess the interactions between biotinylated (Bio)-proteins for AtHAK1 (AH1) or AtPBL27 (PBL) and FLAG-conjugated proteins for *Escherichia coli* dihydrofolate reductase (DR) serving as control, AtCERK1 (AC1), and AtHAK1 proteins. Recombinant proteins synthesized using the cell-free system are presented in Supplementary Fig. 12. All the individual data points are shown with the means and standard errors (*n* = 3). Means indicated by different small letters are significantly different among the respective sets of data, based on a one-way ANOVA with post hoc Tukey’s HSD (*P* < 0.05). **b** AtHAK1 fused to the N-terminal fragment of Venus (nVenus) and AtHAK1 or AtPBL27 fused to the C-terminal fragment of Venus (cVenus) were co-expressed in *Nicotiana benthamiana* leaf cells. **c** FLAG-tagged AtHAK1 (AtHAK1-FLAG) and HA-tagged AtHAK1 (AtHAK1-3HA), AtHAK1-FLAG, AtHAK1-FLAG and HA-tagged AtPBL27 (AtPBL27-3HA) or AtPBL27-3HA were expressed in *Nicotiana benthamiana* leaf cells. Total proteins extracted from the leaves were immunoprecipitated using anti-FLAG-tag magnetic beads, subjected to SDS-PAGE, and probed with anti-HA or anti-FLAG antibody as a primary antibody. Arrowheads indicate the predicted, tagged AtHAK1 or AtPBL27 signals. Source data is presented in Supplementary Fig. 13.

Next we assessed the interaction abilities of AtHAK1 and AtPBL27 molecules, using the AlphaScreen system (Fig. 7a). We found that (i) AtHAK1 formed a homodimer and (ii) similarly to AtCERK1, AtHAK1 was able to interact strongly with AtPBL27. Interactions of those molecules assessed using BiFC assays confirmed interactions between AtHAK1 molecules themselves or AtHAK1 and AtPBL27 along the plasma membrane of *Nicotiana benthamiana* leaves in BiFC assays using the *Agrobacterium tumefaciens*-mediated transient expression system (Fig. 7b). Moreover, immunoprecipitation assays also confirmed in vivo interactions between AtHAK1 molecules themselves or AtHAK1 and AtPBL27 in *N. benthamiana* leaves following their transient expression (Fig. 7c).

Finally, AlphaScreen system and BiFC assays preliminarily resulted in the observation of interactions between GmHAKs and AtPBL27 (Supplementary Fig. 11), implying that AtPBL27 might transmit the signal from the heterozygously expressed soybean HAKs for the defense response in the transgenic Arabidopsis leaves in response to *S. litura* OS (FrA or Frα) (see above).

## Discussion

HDS, including elicitor(s) present in herbivores’ OS, induce host plants’ defense responses mediated by early cellular responses (e.g., Ca^2+^ influx)^32^, hormone signaling (e.g., JA-signaling)^33^, and the activation of defense genes and biosynthesis of defense products^34^. HAKs were characterized here as RLKs that specifically respond to herbivore elicitors but not MD alone (Figs. 1a, 6a, Supplementary Fig. 2). HAKs are predicted to be *Spodoptera* spp-elicitor-specific RLKs (Fig. 1c). Of the HAKs found here, GmHAK1 and AtHAK1 were predicted to be LRR-RLKs (Fig. 1d). Other members of this group, BAK1, FLS2, and EFR, have been clearly shown to respond to pathogenesis peptide elicitors^18^, so it initially seemed possible that GmHAK1 and AtHAK1 might likewise perceive peptides present in or produced by degradation of proteins from *S. litura* OS. Although the ^1^H-NMR analysis of Frα or FrII, which showed minor signals in the aliphatic region (1–2.5 ppm) that were presumed to arise from protein/peptide molecules (Supplementary Figs. 5c, d), it seemed unlikely that GmHAK1 responded to peptides, because heat-denaturation or protease treatment of FrA scarcely suppressed the elicitor activity (Supplementary Fig. 3b). More importantly, given the assumption that GmHAK1 does not interact directly with the elicitor ligand (Supplementary Fig. 8), it is possible that GmHAK1 may be involved in amplification of molecular signals transmitted from the cognate FrA/Frα receptors.

On the other hand, GmHAK2 has a proline-rich sequence in its extracellular domain region (Fig. 1d), and thus this HAK is predicted to be a member of proline-rich extensin-like receptor kinases (PERKs). Fifteen PERK genes are predicted to be present in the Arabidopsis genome, and they may be involved in cell expansion, growth and floral development^35^. To our knowledge, GmHAK2 is annotated as a novel member of the PERK family that is involved in the plant defense system and that may also function as an HDS-transmitting and/or amplifying RLK.

It should be noted that we cannot exclude the possibilility that HAKs may act as partner membrane proteins (i.e., co-receptors) of receptor proteins that interact with Frα directly, as shown for rice OsCERK1 co-receptor, which cooperates with rice CEBiP and LYP4/6 to respond to chitin and peptidoglycan elicitors, as well as for Arabidopsis AtCERK1, which cooperates with LYM1/3, which are peptidoglycan receptors^18,19^. The extracellular domain of OsCERK1 has been shown to promote formation of OsCERK1 homodimer, which interacts with two CEBiP moleules, resulting in a sandwich-like complex, when chitin oligosaccharide elicitor binds to OsCERK1^19^.

Given the fact that the HAKs found here may be composed of the respective homodimers (GmHAK1/GmHAK1, GmHAK2/GmHAK2, or AtHAK1/AtHAK1) (Figs. 3a, 7), we propose a possible model in which HAKs work as co-receptors (like OsCERK1) to transmit the phosphorylation signal to intracellular molecule(s). This accords with the structural characteristic of HAKs, which contain the arginine-aspartate (RD) type of kinase domain. The RD type kinases (e.g., BAK1 and CERK1) are thought to generate the initial phosphorylation signal by interacting with non-RD type receptor kinases (e.g., FLS2, EFR and LYK5) which lack kinase activity but can bind the ligand molecule and then activate the partner co-receptor(s)^19,36^. An alternative possibility is that the ITC system might not be appropriate for evaluating the HAK-Frα interactions or that Frα might be structurally modulated or degraded *in planta* prior to perception by HAKs (see below).

Regarding the partner protein for the intracellular signaling of HAKs, receptor-like cytoplasmic kinases (RLCKs) act as a major class of signal transmitting proteins after elicitor perception in Arabidopsis^37^. AtPBL27 (a member of the RLCKs) contributes to MAPK activation, defense gene activation, and callose deposition, but not to reactive oxygen species (ROS) generation, in chitin signaling in Arabidopsis^19,21^. A BLAST search for AtPBL27 showed about 80% identity of amino acid residues with a suite of soybean PBL27s (e.g., GLYMA15G096800 and GLYMA13G216100), suggesting that the FrA/Frα perception system consists of HAK1 and PBL27 in a variety of plant taxa, including Brassicaceae and Leguminosae.

In Arabidopsis, the involvement of jasmonate signaling, the primary signaling pathway for HDS-inducible defense response^38^, in OS signaling after herbivore attack should also be considered here. The HAK/PBL27 system is not likely to serve as an upstream signal for de novo jasmonate synthesis (Fig. 6c). Instead, the HAK/PBL27 system is more likely to be involved in the activation of ethylene synthesis and signaling, in agreement with our findings that an Arabidopsis mutant line that is ethylene insensitive (*ein2-1*) completely lacks expression of *PDF1.2* in response MD + Frα (Fig. 6d). The same held true for JA-Ile receptor COI1 (*coi1-1*), indicating the involvement of the HAK/PBL27 system in jasmonate signaling but not jasmonate synthesis (Fig. 6d).

Polysaccharides are thought to be major components of Frα (or FrA and FrII) (Supplementary Figs. 2, 5). They are large molecules (>15 kDa; Fig. 2b), and classically characterized low-molecular-weight elicitors, including FAC-type elicitors^6^, peptide-type elicitors such as inceptin^9^, and fatty acid-type elicitors (e.g., caeliferins)^8^ do not appear likely to contribute to the elicitor activity of Frα. However, the possibility that Frα components are degraded by host plant enzymes such as polysaccharide-degrading enzymes, eventually resulting in the generation of low-molecular weight oligosaccharide elicitors that are recognized by PRRs in the host plants, has not been excluded. This possibility might accord with the observation that the ITC system showed no affinities of HAKs with the original molecular components of Frα (Supplementary Fig. 8). It is also likely that polysaccharide-degrading enzymes, including glucosidase and arabinofuranosidase localized at the cell wall/plasmadesmata or herbivory-associated cell component derivatives, may contribute to Frα degradation. Further comprehensive and unified studies on an array of trafficking, perception, amplifying, and transmitting systems for *S. litura*-derived HDS in the extracellular space as well as intracellular signaling for defense responses of plants, should be performed to deepen our understanding of the nature of the HAK-mediated signaling network.

## Methods

### Plants and insects

WT and T-DNA insertion mutant (*athak1* [SALK_134409C], *athak1-2* [GK_958D06], *at5g01950* [SALK_009439], *cerk1* [GABI_096F09]^39^, *atpbl27* [GABI_001C07]^21^, *pub4* [SALK_054373]^40^, *bik1*/*pbl1*^41^, *xlg1/2/3*^42^, *bak1-4* [SALK_116202]^43^), *ein2-1* and transgenic lines of *Arabidopsis thaliana* (Col-0) plants and soybean (*Glycine max*, cv. Enrei) plants were, respectively, grown in soil in climate-controlled rooms at 22 ± 1 °C with a photoperiod of 12 h (80 µE/m/s) and at 24 ± 1 °C with a photoperiod of 16 h (80 µE/m/s). The individual plants were grown in single plastic pots. The potted, 4-week-old Arabidopsis plants and 2-week-old soybean plants were used for subsequent analyses. The *coi1-1* seeds were germinated on 1/2 Murashige and Skoog medium supplemented with 2% sucrose, 0.8% agar, and 50 µM MeJA (Wako Pure Chemical Industrials, Ltd., Osaka, Japan) to screen for the individuals showing normal root growth for 2 weeks. The screened plants were transferred and grown in plastic pots for an additional 3 weeks for the assay^44^ shown in Fig. 6d.

Eggs of *S. litura* (Fabricius) were obtained from Sumika Technoservice Co. Ltd. (Takarazuka, Japan). They were incubated in a climate-controlled room at 24 ± 1 °C with a photoperiod of 16 h. The hatched larvae were reared on artificial diet (Insecta LFS, Nihon Nosan Kogyo Ltd., Tokyo, Japan) in a plastic container (0.9 L) with a mesh-covered lid. Feces in the plastic case were removed and a piece of artificial diet was added three times a week. When larvae reached the final instar, they were transferred to a larger container (1.6 L) with a mesh-covered lid. Finally, about 30 pupae of *S. litura* were transferred to a lidded plastic cylinder (ø 12 cm × 33 cm) and reared until their adult stage. Meanwhile, their oviposition was allowed on a piece of paper that completely covered the bottom of the cylinder. The eggs and larvae were used for assays or for continuous rearing on artificial diet (see above), except for the larvae reared after hatching for 1 week on fresh Arabidopsis or soybean plants in the laboratory at 24 ± 1 °C and used for analyses shown in Supplementary Fig. 7.

Adults of *P. rapae* were collected in a field in Katsushika-ku, Tokyo. The larvae were hatched and reared on *Brassica rapa* var. *perviridis*.

### Primers

Primers used for all the polymerase chain reactions (PCRs) in this study are listed in Supplementary Table 2.

### Transgenic Arabidopsis plants of soybean RLKs

The full-length ORFs of soybean PRR homologs were obtained from Legume Base NBRP (https://www.legumebase.brc.miyazaki-u.ac.jp/). The ORF of each clone was amplified using KOD Plus Neo (Toyobo, Osaka, Japan) and a pair of specific primers (Supplementary Table 2) and inserted into binary vector pMDC32 (2x 35SP::Gateway (GW) region::*nopaline synthase* terminator [NOST]) using the Gateway cloning system (Thermo Fisher Scientific, Waltham, MA, USA). The resulting vector pMDC32-GmHAK was transformed into *A. tumefaciens* strain EHA105 by electroporation. Col-0 WT Arabidopsis plants that had been grown for 6–7 weeks were transformed via the floral-dip transformation method and selected^45^. T_3_ homozygous plant lines were used for further analyses.

### SEC of OS

Larvae were reared on artificial diet or fresh Arabidopsis plants (about 4 weeks old) or soybean plants (see above). OS was collected from third or fourth instar larvae of *S. litura* using a glass capillary tube (Hirschmann Laborgeräte GmbH & Co. KG, Eberstadt, Germany). *M. loreyi* OS was previously obtained in Okayama University^46^. The collected OS was stored at −20 °C until use.

To carry out SEC, crude OS was lyophilized using an FDU-1200 freeze dryer (EYELA, Tokyo, Japan). About 150 mg of lyophilized OS was dissolved with 3 mL of 50 mM ammonium bicarbonate (AMBIC) buffer (pH 8.0). For the first-round fractionation, OS was passed through a column (1.5 cm × 98.5 cm) packed with Bio-Gel P-2 resin (Bio-Rad, Hercules, USA), and 1.5 mL fractions were collected (yielding FrA to C). All of the fractions were lyophilized and then dissolved with 150 µL AMBIC buffer for the measurement of refractive index (10-fold concentrated) or with 750 µL of 10 mM MES buffer (pH 6.0) for assays (2-fold concentrated). FrA (about 70 mg) was purified through a Bio-Gel P-10 resin (Bio-Rad) column (2.0 cm × 45 cm). Fractions (1.5 mL) were collected during the course of elution (yielding Frα to γ). Again, all the fractions were lyophilized and then dissolved with 150 µl of AMBIC buffer for the measurement of refractive index (10-fold concentrated) or with 750 µl of 10 mM MES buffer (pH 6.0) for assays (2-fold concentrated). The temperature-corrected refractive index of fractions was monitored using a Brix/RI-Chek Refractometer (Reichert, NY, USA).

### Characterization of FrA/Frα

For protease treatment of FrA, 3 mg of the lyophilized fraction were dissolved in 50 mM AMBIC buffer (pH 8.0) and digested with 1% (w/w) protease XIV (Sigma-Aldrich, St. Louis, MO, USA) for 24 h at 37 °C to degrade proteins, and boiled at 80 °C for 20 min to deactivate proteases.

The purification trials of FrA were performed using the CombiFlash Rf purification system (Teledyne ISCO, Lincoln, NE, USA). Pre-packed cartridges, RediSep Rf Gold C18Aq (Teledyne ISCO) and RediSep Rf Gold Diol (Teledyne ISCO) were used, respectively, for reversed- and normal-phase chromatography. The reversed-phase column (5.5 g media) was previously conditioned with phase A (pure water) and eluted with a 5.7-min linear gradient from 0 to 100% phase B (acetonitrile). For the normal-phase chromatography, the column (5.5 g media) was previously conditioned with phase A (acetonitrile) and eluted with a 5.7-min linear gradient from 0 to 100% phase B (pure water).

For proton nuclear magnetic resonance (^1^H-NMR) analysis, a lyophilized Frα or FrII sample (5 mg) was dissolved in 0.7 mL of D_2_O, and the spectrum was recorded using a Bruker Avance DRX-600 spectrometer (Bruker, Billerica, MA, USA) at 298 K.

Sugar components and the linkage pattern of monosaccharides in Frα were determined by the acetylated methyl glycosides method^27^ and the partially methylated alditol acetates method^28^, respectively. Monosaccharide derivatives were detected using the GCMS-QP2010 Plus GC-MS system (Shimadzu, Kyoto, Japan) with an HP-5MS capillary column (Agilent Technologies, Santa Clara, CA, USA). The carrier gas was He, with a 10 mL/min flow, injection volume 1 µL in acetone, injection port 220 °C with 1:2.5 split ratio. GC-oven temperature was programmed to rise from 40 °C (2 min hold) to 280 °C (10 min hold) at 10 °C/min. Electron ionization spectrometer range was 45–500 *m*/*z*.

### OS and herbivore treatment

All of the crude OS and the fractionated OS samples were diluted 3- and 5-fold with 10 mM MES buffer (pH 6.0), respectively. Chitin oligosaccharide [(GlcNAc)_7_] was diluted to 10 µM with 10 mM MES buffer (pH 6.0). 18:3-Gln was diluted to 100 µM in 10 mM MES buffer (pH 6.0) with 0.01% Tween20. Mechanical damage (MD) was performed with stainless steel needles on 4 leaves of an individual Arabidopsis plant and one of the secondary leaves of individual soybean plants. Approximately 30 MD spots were made per leaf. The crude OS, the fractionated OS, chitin oligosaccharide or 18:3-Gln solution was immediately applied onto an MD spot (~1 µL per spot). Treatment of MD leaves with MES buffer served as a control.

### RNA extraction, cDNA synthesis, and quantitative PCR

Approximately 100 mg of leaf tissues were homogenized in liquid nitrogen, and total RNA was isolated and purified using Sepasol®-RNA I Super G (Nacalai Tesque, Kyoto, Japan) following the manufacturer’s protocol. Single-stranded cDNA was synthesized using ReverTra Ace qPCR RT Master Mix with gDNA Remover (Toyobo), and 0.5 µg of the total RNA was incubated, first at 37 °C for 5 min for the DNase reaction and then at 37 °C for 15 min for the RT reaction. Real-time PCR was performed using a CFX Connect real*-*time PCR detection system (Bio-Rad) with THUNDERBIRD SYBR qPCR Mix (Toyobo) and gene-specific primers (Supplementary Table 2). The following protocol was used: an initial polymerase activation of 60 s at 95 °C, followed by 45 cycles of 15 s at 95 °C and then 30 s at 60 °C. Then, a melting curve analysis preset by the instrument was performed^10^. Relative transcript abundances were determined after normalization of raw signals with the abundance of the housekeeping transcript of the Arabidopsis *ACT8* gene (*at1g49240*) or soybean *ACT* gene (*GQ339774.1*). We did not use samples or data when sufficient amounts or quality of RNA (> 83 ng/µL) were not obtained from leaves or when abnormal quantification cycle (Cq) values for the actin gene were obtained.

### Subcellular localization of recombinant GmHAK proteins

The full-length ORFs of *GmHAK1* and *GmHAK2* (without stop codon) were cloned in Gateway (GW) vector pGWB451 or pGWB405 (35SP::GW::GFP::NOST)^47^, resulting in the construction of vectors pGWB405-GmHAK1-GFP and pGWB451-GmHAK2-GFP. These vectors, plus pGWB452 (35SP::GFP::GW), which served as a control, were transiently transformed into onion epidermal cells. The cells were bombarded with 15 µg of vector coated onto 3 mg of tungsten particles (Rare Metallic Co., LTD, Tokyo, Japan) using the PDS-1000/He particle delivery system (Bio-Rad). Onion cells were incubated in the dark at 28 °C for 24 h after bombardment, and GFP fluorescence was observed under a fluorescence microscope BZ-X700 (Keyence Co., Osaka, Japan). For plasmolysis, the sample was treated with 0.6M mannitol for 15 min before observation.

### Preparation of recombinant GmHAK proteins and ITC analysis

The extracellular domain of GmHAK1 (amino acid residues 24–556) and the full-length ORF of *GFP* were subcloned into pET23a. Similarly, the extracellular domain of GmHAK2 (amino acid residues 1–159) was subcloned into pDEST17. The recombinant vectors were transformed into *Escherichia coli* BL21-CodonPlus(DE3) to prepare the recombinant proteins. The proteins were extracted and purified using Ni Sepharose High Perfomance (GE Healthcare, Buckinghamshire, UK) following the manufacturer’s protocol. The protein was further purified with a gel filtration chromatography column, HiLoad 26/60 Superdex 200 (GE Healthcare), equilibrated with buffer (20 mM HEPES buffer (pH 8.0), 300 mM NaCl, 10% glycerol). Fractionated protein was concentrated with Amicon Ultra-15 (Merck Millipore Ltd, Darmstadt, Germany). Each of the boiled proteins in the sample buffer was subjected to 14% sodium dodecyl sulfate (SDS)–polyacrylamide gel electrophoresis (PAGE).

Molecular interaction analysis between recombinant GmHAK proteins and Frα was performed using the MicroCal iTC_200_ system (Malvern Panalytical, Malvern, UK). Frα (2.5 mg/mL) was injected through the computer-controlled microsyringe (40 μL) at an interval of 2 min into the sample cells (cell volume = 200 μL) filled with recombinant GmHAK1 (25 µM) or GmHAK2 (50 µM) or GFP reference protein (50 µM) while stirring at 1000 rpm. The entire experiment was conducted at 30 °C. All ITC data were controlled by subtraction of the reference experimental data that were obtained for the titration of Frα against protein-free buffer. The data obtained were analyzed and the figure was prepared using the Origin 7.0 software package provided by Malvern Panalytical.

### Cell-free protein synthesis, immunoblotting, and AlphaScreen system

The full-length ORFs of *GmHAK1*, *GmHAK2*, *AtHAK1, CERK1*, and *AtPBL27* were inserted into the GW vector pEU-GW-AGIA, pEU-GW-bls (bls; biotin ligation site), or pEU-GW-FLAG for cell-free protein synthesis, resulting in the construction of vectors pEU-GmHAK1-AGIA, pEU-GmHAK2-AGIA, pEU-GmHAK1-bls, pEU-GmHAK2-bls, pEU-AtHAK1-bls, pEU-AtPBL27-bls, pEU-CERK1-FLAG, pEU-AtHAK1-FLAG, pEU-GmHAK1-FLAG, and pEU-GmHAK2-FLAG. Cell-free protein synthesis, AlphaScreen-based protein–protein interaction assays, and immunoblotting were carried out according to the methods described previously^48^. Frα was added at 0, 0.4, 4, or 40 µg/mL. For quality checking of the proteins used, total proteins were subjected to 10% SDS-PAGE and immunoblotted with AGIA HRP-linked antibody^48^, anti-Biotin HRP-linked antibody (Cell Signaling Technology, Beverly, MA, USA), or monoclonal anti-FLAG M2-peroxidase antibody produced in mouse clone M2 (Sigma-Aldrich).

### BiFC assays

The full-length ORFs of *GmHAK1*, *GmHAK2*, *AtHAK1*, and *AtPBL27* (without stop codon) were inserted into the GW vector pDEST-GW-nVenus, along with the N-terminal fragment of Venus, and pDEST-GW-cVenus, along with the C-terminal fragment of Venus, respectively, resulting in the construction of vectors 35SP-GmHAK1-nVenus, 35SP-GmHAK2-nVenus, 35SP-GmHAK1-cVenus, and 35SP-GmHAK2-cVenus. Each pairwise combination of vectors (15 µg of each vector) was transiently co-transformed into onion epidermal cells using the PDS-1000/He particle delivery system (see above).

For the *A. tumefaciens*-mediated transient expression system using *N. benthamiana* grown for 3 weeks, a pair of *A. tumefaciens* EHA105 carrying the indicated vectors 35SP-AtHAK1-nVenus, 35SP-GmHAK1-nVenus, or 35SP-GmHAK2-nVenus, together with 35SP-AtHAK1-cVenus or 35SP-AtPBL27-cVenus, were pressure-infiltrated into the leaves of *N. benthamiana* as reported^40^.

### Virus-induced gene silencing

cDNA fragments of *GmHAK1* (162 bp) and *GmHAK2* (180 bp), were amplified by PCR using primer pairs containing XhoI and BamHI sites (Supplementary Table 2) and ligated to pEALSR2L5R5^29^, resulting in the construction of pEALSR2L5R5-GmHAK1 and pEALSR2L5R5-GmHAK2, respectively. Viral inoculation of soybean plants was carried out according to the method described previously^29^. Briefly, *Chenopodium quinoa* was inoculated with 20 µg of vector mixture (pEALSR1, pEALSR2L5R5-GmHAK1, or pEALSR2L5R5-GmHAK2) by mechanical wounding using carborundum. RNA was extracted from virus-infected *C. quinoa* leaves, and 400 µg of total RNA was coated onto 16 mg of tungsten particles. Soybean cotyledons were bombarded 10 times at 1100 psi using a PDS-1000/He particle delivery system (see above). The plants were grown in soil for 3 weeks and used for analyses.

### Herbivore assay

A third-instar larva of *S. litura*, starved overnight, was released onto one of the secondary leaves of a potted soybean plant. The leaf was covered with a mesh bag and kept for 24 h. The leaves were then scanned, and the total leaf area and the consumed leaf area were determined using ImageJ. Replicate analyses were conducted with 9–11 independent samples.

We also performed assays to assess the growth of *S. litura* larvae on Arabidopsis plants. Third-instar larvae were initially weighed (1.8–2.0 mg), and each larva was released onto a potted plant. The net body weight that *S. litura* larvae gained at 22 ± 1 °C (14 h photoperiod at 80 µE/m/s) during 2 and 4 days after they had been released onto the plants was determined. When a larva was dead or lost during assays, we excluded that sample, and final replicate analyses were conducted with 12 independent samples^44^.

### Phytohormone analysis

Phytohormone levels of leaves subjected to MD alone or MD + Frα treatment or of untreated leaves were determined. The jasmonate levels of leaves (60–100 mg fresh weight) were determined according to the method described previously^49^, with slight modifications.

Ethylene was measured in the headspace (45 mL) of 15 cut mature leaflets prepared from 3 plants. Ethylene was allowed to accumulate for 24 h at normal photoperiod, after which 1 mL of headspace air was removed by syringe with needle inserted through a silicon plug. Air samples were introduced to gas chromatograph GC-2014 (Shimadzu) equipped with Shincarbon ST stainless steel column (length 2 m; ID 3.0 mm; SHINWA Chemical Industries, Ltd., Kyoto, Japan) via manual injection port kept at 200 °C. Detector was flame ionization (FID) held at 210 °C. Column was kept at constant temperature 200 °C and helium flow 50 mL/min. Peak area was compared with ethylene concentration obtained from external 0–1 ppm calibration curve of ethylene standard (GL Sciences Inc., Tokyo, Japan).

### Co-immunoprecipitation assay

The full-length ORFs of *AtHAK1* and *AtPBL27* (without stop codon) were inserted into the GW vector pGWB11 (35SP::GW::FLAG) or pGWB14 (35SP::GW::3xHA). As in BiFC assays, *A. tumefacience* EHA105 carrying each vector was pressure-infiltrated into the leaves of *N. benthamiana*^40^. After 2 days, total proteins were extracted with extraction buffer (50 mM Tris-HCl (pH 7.5), 150 mM NaCl, 10% glycerol, 5 mM DTT, 2 mM EDTA, 1 mM NaF, 1 mM Na_2_MoO_4_–2H_2_O, 0.5% polyvinylpyrolidone, 1% NP-40, and Complete Protease Inhibitor Cocktail tablets (Roche Applied Science, Indianapolis, IN, USA)). Extracted proteins were incubated overnight with anti-DYKDDDDK-tag antibody magnetic beads (#017-25151, Wako Pure Chemical Industrials, Ltd.) at 4 °C, and the beads were washed four times with TBS containing 0.5% NP-40. Immunoprecipitates were eluted with Laemmli SDS sample buffer and used for 10% SDS-PAGE. Anti-FLAG antibody (#F3165, Sigma-Aldrich) and anti-HA antibody (#11867423001, Roche Applied Science) were sued as the primary antibodies. Horseradish peroxidase-linked anti-mouse antibody (#7076, Cell Signaling Technology) or anti-rat antibody (#7077, Cell Signaling Technology) was used as the secondary antibody. The membranes were soaked with Immobilon Western Chemiluminescent HRP Substrate (Merck Millipore) and the signals were detected with an ImageQuant LAS-4000 imaging system (GE Healthcare).

### Statistics and reproducibility

We performed one-way ANOVA with Holm’s sequential Bonferroni post hoc test and post hoc Tukey’s HSD using the program (http://astatsa.com/OneWay_Anova_with_TukeyHSD/) for comparing multiple samples. The sample sizes and number of replicates for all the sets of assays and analyses are indicated in the legends of the corresponding Figures.

### Reporting summary

Further information on research design is available in the Nature Research Reporting Summary linked to this article.

## Supplementary information

Supplementary Data 1

Reporting summary

Supplementary Information

## Data Availability

The source data presented in figures and Supplementary Table 1 are available in Supplementary Data 1.
