# Supplementary material for: Soy and Arabidopsis receptor-like kinases respond to polysaccharide signals from Spodoptera species and mediate herbivore resistance
Source: Commun Biol. 2020 May 8;3:224. doi: 10.1038/s42003-020-0959-4 (PMC7210110; doi:10.1038/s42003-020-0959-4)
Supplement: Supplementary file 3 — Supplementary Information [file 42003_2020_959_MOESM3_ESM.pdf]

Supplymentary Table 1: Relative expression levels of RLK genes in transgenic Arabidopsis leaves

Line	Clone name	Accession number	Conserved motif of ectodomain	mean*	SE*
R1	GMFL01-36-K12	AK245648	RCC1	4.260	2.608
R2	GMFL01-06-C24	AK244534	GUB_WAK_bind	6.830	1.564
R3	GMFL01-06-N22	AK244566	EGF/GUB_WAK_bind	34.762	3.324
R4	GMFL01-05-J08	AK285234	LRR	0.025	0.003
R5	GMFL01-30-D17	AK245428	LRR	0.015	0.008
R5-2				0.006	0.003
R6	GMFL01-36-D09	AK286757	Malectin	0.239	0.035
R7	GMFL01-26-P11	AK245323	Malectin	0.058	0.024
R8	GMFL01-38-G21	AK286845	Malectin	4.382	0.520
R9	GMFL01-01-N11	AK244005	not found	2.531	0.127
R10	GMFL01-40-A08	AK245733	not found	8.386	0.639
R11	GMFL01-01-J09	AK243908	not found	4.362	0.350
R12	GMFL01-43-N13	AK245867	Lectin	46.551	6.410
R13	GMFL01-39-B18	AK286868	Lectin	2.312	0.764
R14	GMFL01-17-D12	AK285833	PRICHEXTENSN	0.004	0.001
R14-2				0.003	0.000
R15	GMFL01-02-E18	AK244175	USP	2.445	0.264

Relative transcript abundances were determined after normalization of raw signals with the abundance of the housekeeping transcript of an *ACT8* gene (*at1g49240*). All the individual data points are shown with the means and standard errors (n = 4-6).

Conserved motifs were predicted using an InterPro (http://www.ebi.ac.uk/interpro/).
EGF, epidermal growth factor-like domain; GUB_WAK_bind, wall-associated receptor kinase galacturonan-binding; LRR, leucine-rich repeat; PRICHEXTENSN, proline rich extensin signature; RCC1, regulator of chromosome condensation 1; USP, universal stress protein family.

Supplementary Table 2: Primers

Purpose	Plant	Gene (GAI code)	Forward primer sequence (5' to 3')	Reverse primer sequence (5' to 3')	Remarks
RT-qPCR	Arabidopsis	ACT8 (At1g49240)	TGCTGGTCGTGACCTTACTG	CGAGGTCTCCATCTCTTGCT	
		PDF1.2 (At5g44420)	CGAACATGGTGTTTGTGAGG	ATGTGCCATTCGAGCAAGAT	
		TI (At2g43510)	GTGATGTTGGTTTCGGCTTT	CTGTGGAGTATCGTCGCAGA	
		AtHAK1 (At1g06840)	ATGGCAGGAAAGAGAGCTGA	GGTGGGCAGTCAGAACAAAT	
		At5g01950	TTTGGCACTGAGGTGTAGCC	TTCGACGACGATGTTGACAG	
	Soybean	ACT (GQ339774.1)	CATCCAGGCTGTTCTCTCCT	CAGCGAGATCCAAACGAAGG	
		PR (HQ913577)	CGGAGCCATTGGAGAAGATA	GCAGATCTCTCCTTGCCAGT	
		TI (AF128268.1)	TTTCCCTTTCCTTCCTGCCT	TCCGGCCTAGTTTCAATCCA	
		R1 (AK245648)	CAGTGGCCTACGTCGGTTAT	GAATCGGTGGTGGACCTAGA	
		R2 (AK244534)	AGGTGTTGGAGGCTATGGTG	AGGTTGCGGTGGTTAACTTG	
		R3 (AK244566)	GTTCGCCTCTTAGGTTGCTG	GCAGTTTCGGTAGCAATGGT	
		R4 (AK285234)	AAACCAAGGACCGAAGGAGT	TTGCTTCGAGAAATGGCTCT	
		R5 (AK245428)	CCTTGGCTGTTTCGTTTTGT	GCTGCACATGGATCTCCTTT	
		R6 (AK286757)	AAGGCAGCATTGGGTATTTG	GCGACACCTGTTTCTTCTCC	
		R7 (AK245323)	CATAGCTGAAAGCCGTGTCA	TGGAAGAAGAGGCGAAGAAA	
		R8 (AK286845)	TCATCATCGCTACCAACCAA	ACGGGTGTTGAACTGTCCTC	
		R9 (AK244005)	ATCCAGCGGCTTCTGATTTA	CCATTTCCTCGTGCAGGTAT	
		R10 (AK245733)	CCTGAGGGGATGACTCACAT	CGGTTTGAAGTGCCTTTCTC	
		R11 (AK243908)	TGTGGCTAAGGGTCTTGCTT	TCCCTCATTACCTTGCTTGG	
		R12 (AK245867)	GCTTTCTAACGGCGCAATAG	ATCTGTGCGGCCAGTACTCT	
		R13 (AK286868)	ATCCCTTCATTCCCCGTAAC	TCTTTCCGAGGTTTTTGTCG	
		R14 (AK285833)	ACACGAGCTTTGGAAGAGGA	AACAACCTGGCTCATCCTTG	
		R15 (AK244175)	CTCTTGATGGGGTGAGTGGT	ATAATCGGCACAAGGACGAG	
		GLYMA11G200300	ATCTTTCAAAGAGTTTCAAG	AATCAAACGCCTCTTAATGG	
		GLYMA07G004700	ATAGGGGCCGTGTTAGGTTC	CTGAAGTGTGCCACCAGCTA	
Gene cloning		R1	CCACCCACCACCACCAATGGCACCTTTTCTCATTAA	TCCAGCACTAGCTCCAGACTATTGTGAAGAAATAGAAT	underline: S1- or T1-linker sequences
		R2	CCACCCACCACCACCAATGACGACCAGACTCTTCCT	TCCAGCACTAGCTCCAGACTAGTTCTCCACTACCTTTG	underline: S1- or T1-linker sequences
		R3	CCACCCACCACCACCAATGTTTATTCTTCGTCACCA	TCCAGCACTAGCTCCAGATTAGTAGATTTTATGTCTCT	underline: S1- or T1-linker sequences
		R4	CCACCCACCACCACCAATGAACTCCCTAAAATACTT	TCCAGCACTAGCTCCAGATTAACTAACTAGGTTCAATT	underline: S1- or T1-linker sequences
		R5 (GmHAK1)	CCACCCACCACCACCAATGCCGGCTCTGAGAATTCA	TCCAGCACTAGCTCCAGATCAGCGAGGAACAACGGTGG	underline: S1- or T1-linker sequences
				TCCAGCACTAGCTCCAGAGCGAGGAACAACGGTGGGAA	without stop codon; underline: T1-linker sequences
		R6	CCACCCACCACCACCAATGCCAAGTGATTTGTATTA	TCCAGCACTAGCTCCAGATCAACGTGGCTTTGGATCCA	underline: S1- or T1-linker sequences
		R7	CCACCCACCACCACCAATGGACACCACTGACACACG	TCCAGCACTAGCTCCAGATTATCGTCCCTTTGGATCCT	underline: S1- or T1-linker sequences
		R8	CCACCCACCACCACCAATGTGTGCTGCTTCCATTGC	TCCAGCACTAGCTCCAGATCATCGTCCTTTTGGATCCT	underline: S1- or T1-linker sequences
		R9	CCACCCACCACCACCAATGAAGTGTTTTCCATTCTC	TCCAGCACTAGCTCCAGATCAACATGTTCTTACAAGCT	underline: S1- or T1-linker sequences
		R10	CCACCCACCACCACCAATGTCCCTCCCTTTCTATTA	TCCAGCACTAGCTCCAGATCAATCCGTGGTCACAGTCT	underline: S1- or T1-linker sequences
		R11	CCACCCACCACCACCAATGGATAAGCAAATTTTGTT	TCCAGCACTAGCTCCAGACTATACTGTG CTCATCCTTG	underline: S1- or T1-linker sequences
		R12	CCACCCACCACCACCAATGTCTCATTCCAAAACCCC	TCCAGCACTAGCTCCAGATCAAACCGGAGGAGGGGAGG	underline: S1- or T1-linker sequences
		R13	CCACCCACCACCACCAATGAATTTCTTAGCTCTATC	TCCAGCACTAGCTCCAGATTAGCGTCCAACACTTTGAA	underline: S1- or T1-linker sequences
		R14 (GmHAK2)	CCACCCACCACCACCAATGTCAACTGCCCCCGCGCC	TCCAGCACTAGCTCCAGATCAAGAGCTTCCACTGAAAC	underline: S1- or T1-linker sequences
				TCCAGCACTAGCTCCAGAAGAGCTTCCACTGAAACCTT	without stop codon; underline: T1-linker sequences
		R15	CCACCCACCACCACCAATGATGTTAATCGGCGACGG	TCCAGCACTAGCTCCAGATTAGTCAAAACTTGAAGATC	underline: S1- or T1-linker sequences
		AtHAK1	CCACCCACCACCACCAATGTTTTCGACCCATCATGT	TCCAGCACTAGCTCCAGATCTAGGTGCAACTGAGGGAG	without stop codon; underline: S1- or T1-linker sequences
			GGGGACAAGTTTGTACAAAAAAGCAGGCTTCCACCCACCACCAAT	GGGGACCACTTTGTACAAGAAAGCTGGGTCTCCAGCACTAGCTCCAGA	2nd GW-primers; underline: S1- or T1-linker sequences
E.coli protein synthesis		pET23a	CACCACCACCACCACCACTGA	ATGTATATCTCCTTCTTAAA	inverse PCR primers
		GFP	GAAGGAGATATACATATGAGTAAAGGAGAAGAACT	GTGGTGGTGGTGGTGTTTGTATAGTTCATCCATGC	underline: adapter sequence for infusion cloning
		GmHAK1	GAAGGAGATATACATATGTCACTACCAACAGACCCTTC	GTGGTGGTGGTGGTGTTTTATGCCCACATTATTCT	underline: adapter sequence for infusion cloning
		GmHAK2	CCACCCACCACCACCAATGTCAACTGCCCCCGCGCC	TCCAGCACTAGCTCCAGACTACGAAATACTCGACGAGG	underline: S1- or T1-linker sequences
Cell-free protein synthesis		pEU-GW-AGIA, pEU-GW-bls, pEU-GW-FLAG	GCGTAGCATTTAGGTGACACT	CCTGCGCTGGGAAGATAAAC	"Split-Primer" PCR
VIGS		GmHAK1	AACTCGAGAGTGGGGGTGAAATTCCTT	TTGGATCCTGATGGTATGGGTCCAGTGA	underline: XhoI or BamHI site
		GmHAK2	AACTCGAGCCGTCCACTCCTTCTGCTTC	TTGGATCCCGATCCGGACGGAGGAGAGG	underline: XhoI or BamHI site

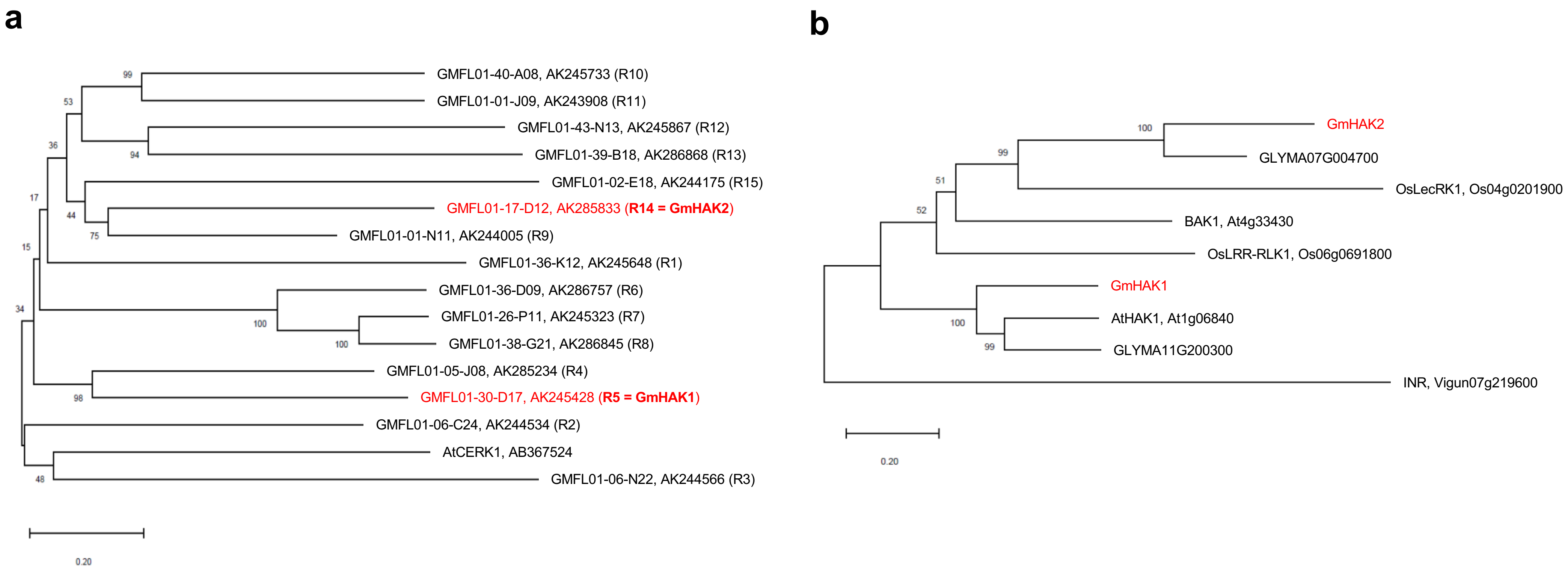

Supplementary Figure 1: Phylogenetic tree inferred from deduced amino acid sequences of the soybean RLKs selected as research targets (**a**) and GmHAK candidates and other RLK proteins that have been reported to serve as herbivore-derived danger signals (**b**) in several plant taxa. Phylogenetic tree was generated by MEGA5 software (https://www.megasoftware.net/), using the neighbor-joining method.

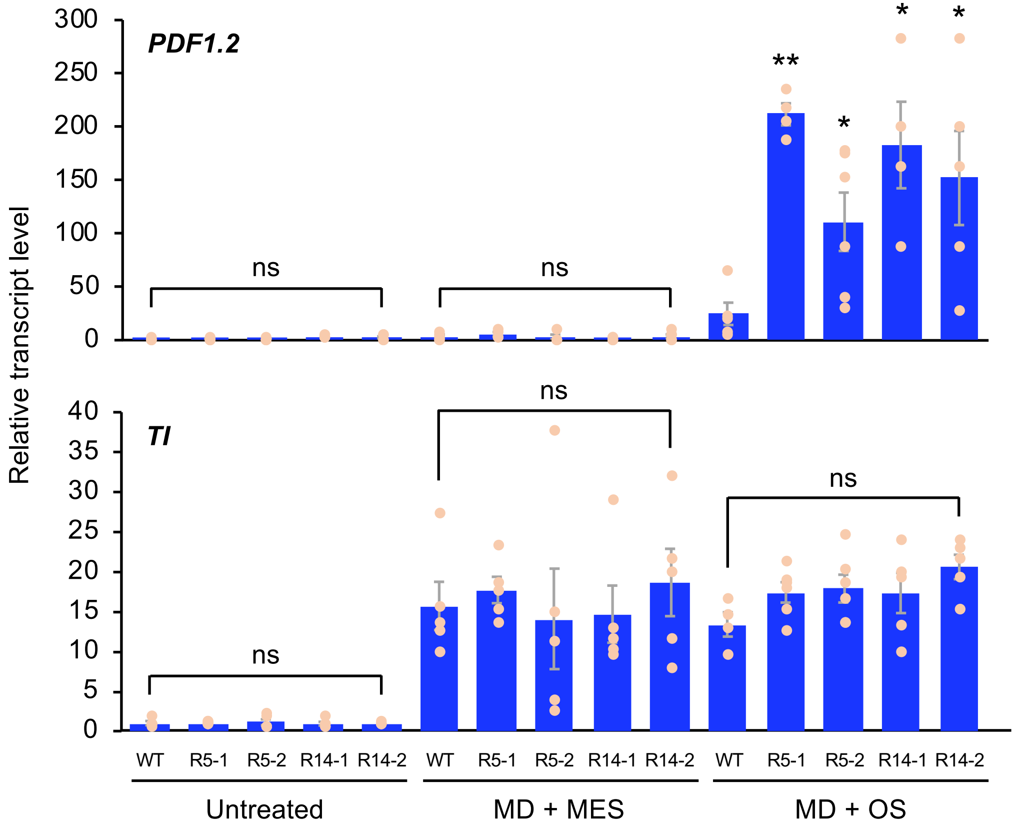

Supplementary Figure 2: Transcript levels of the *PDF1.2* and *trypsin inhibitor* (*TI*) genes in leaves of wild-type (WT) and GmHAK1- or GmHAK2-overexpression lines (R5-1, R5-2, R14-1, and R14-2) of Arabidopsis plants at 24 h after mechanical damage (MD) with or without application of OS from *Spodoptera litura*. All the individual data points are shown with the means and standard errors (n = 4-6). Data marked with an asterisk are significantly different from those of WT, based on an ANOVA with Holm's sequential Bonferroni post hoc test (**, $0.001 \leq P < 0.01$; *, $0.01 \leq P < 0.05$). ns, not significant.

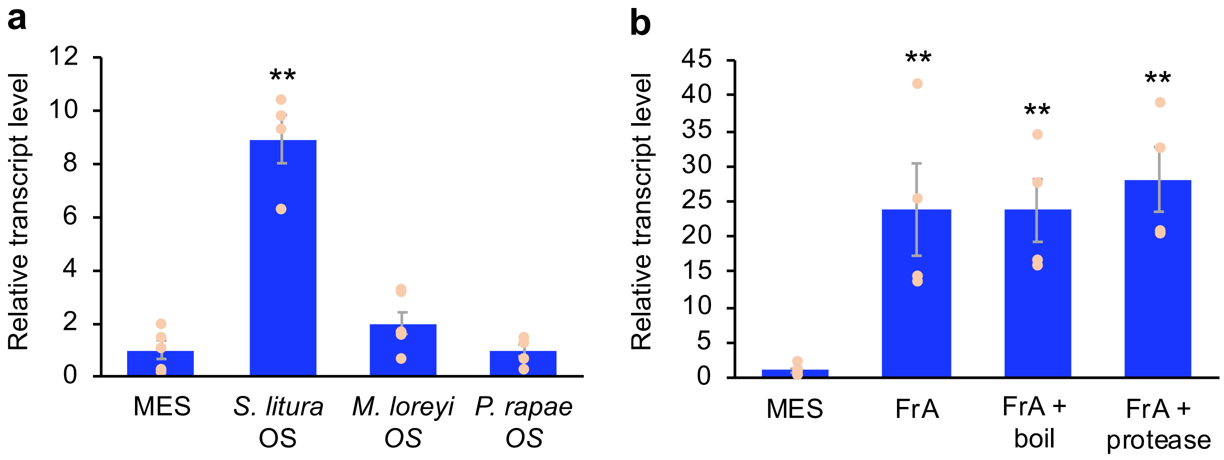

Supplementary Figure 3: Transcript levels of *PDF1.2* in leaves of wild-type (WT) Arabidopsis plants at 24 h after treatment by mechanical damage with application of OS collected from *Spodoptera litura*, *Mythimna loreyi* or *Pieris rapae* larvae (**a**) as well as boiled and protease-treated OS from *S. litura* (**b**). Treatment of mechanically damaged WT leaves with MES buffer served as control. All the individual data points are shown with the means and standard errors (n = 4-6). Data marked with an asterisk are significantly different from those of MES treatment, based on an ANOVA with Holm's sequential Bonferroni post hoc test (**, $0.001 \leq P < 0.01$).

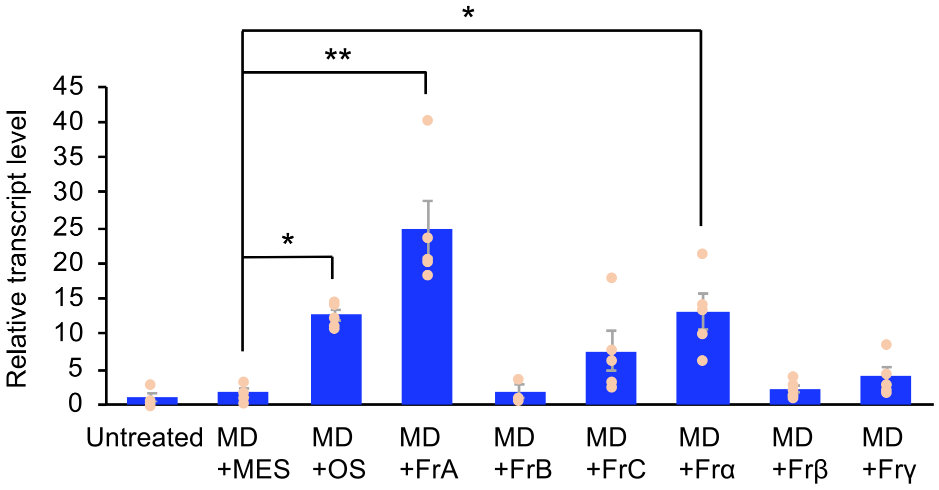

Supplementary Figure 4: Transcript levels of *pathogenesis-related* (*PR*) gene in soybean leaves 24 h after mechanical damage (MD) with application of MES buffer or individual OS fractions (see Fig. 2). All the individual data points are shown with the means and standard errors (n = 3-6). Data marked with an asterisk are significantly different from those of MES treatment, based on an ANOVA with Holm's sequential Bonferroni post-hoc test (**, $0.001 \leq P < 0.01$; *, $0.01 \leq P < 0.05$).

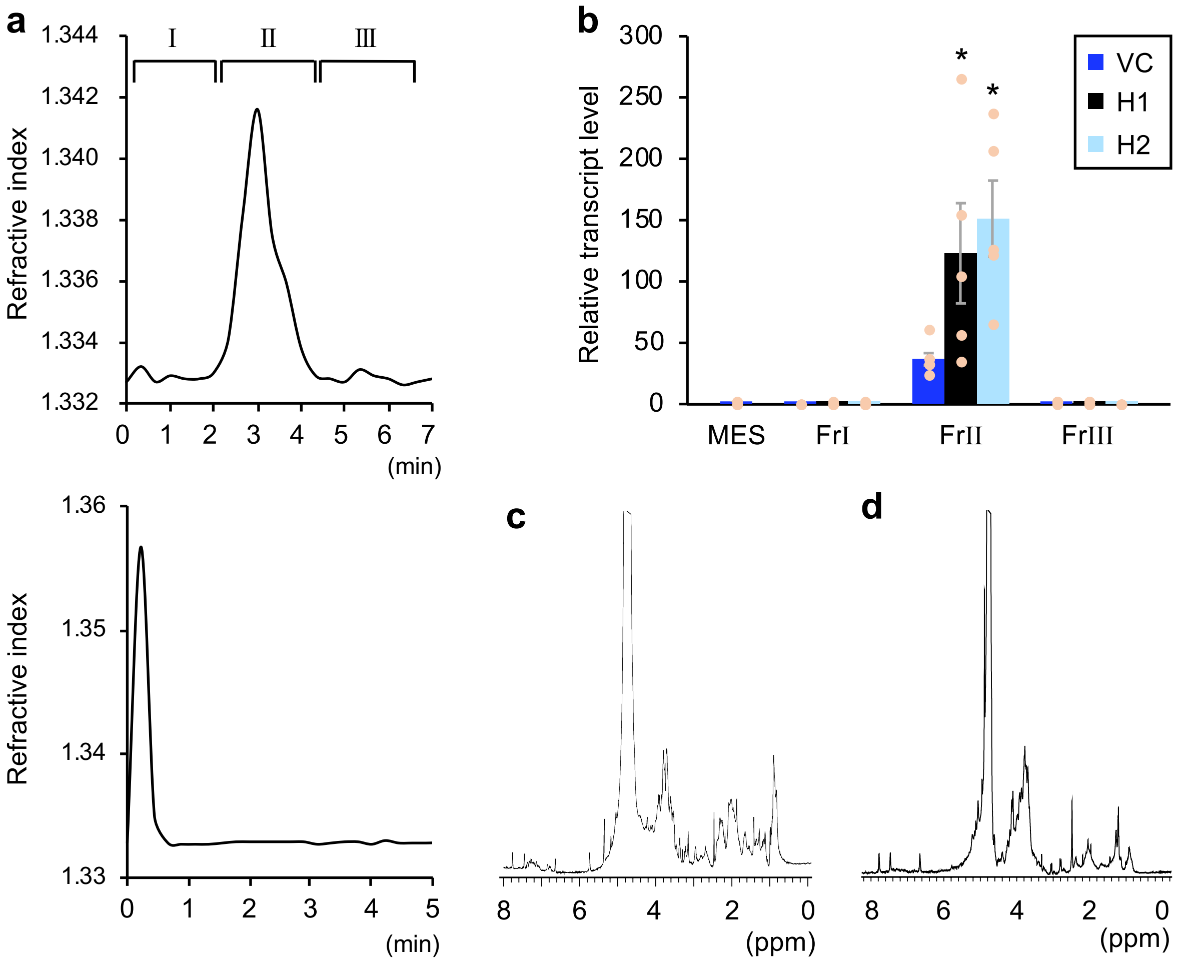

Supplementary Figure 5: Chemical properties of FrII. a Fractionation of FrA, using normal phase chromatography (top) and reverse phase chromatography (bottom). **b** Transcript level of *PDF1.2* in leaves of vector control (VC), and H1 and H2 lines at 24 h after mechanical damage with application of MES buffer or individual OS fractions obtained by normal phase chromatography. All the individual data points are shown with the means and standard errors (n = 5-6). Data marked with an asterisk are significantly different from those of VC, based on an ANOVA with Holm's sequential Bonferroni post hoc test (*, $P < 0.05$). **c, d** ^{1}H NMR spectrum (600 MHz, D_2O) of FrII from normal phase chromatography (**c**) in comparison to that of Frα from size exclusion chromatography (**d**).

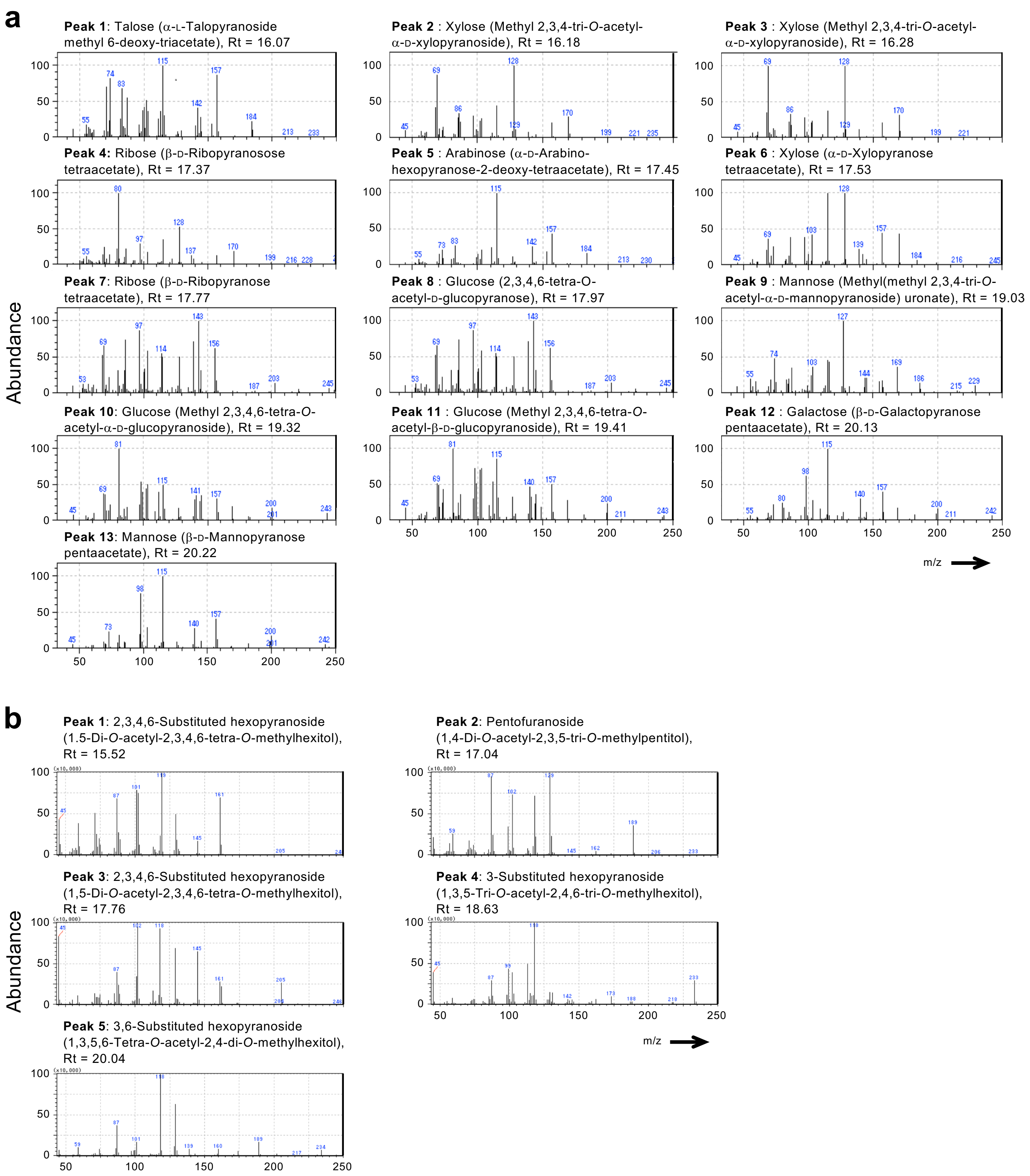

Supplementary Figure 6: The fragmentation patterns of GC-MS mass spectrum from the data for monosaccharide determination analysis of Frα (**a**) and monosaccharide branching point determination analysis of Frα (**b**) (see Figs. 2c and 2d, respectively). The peaks marked with numbers indicate the acetylated methyl glycoside derivatives.

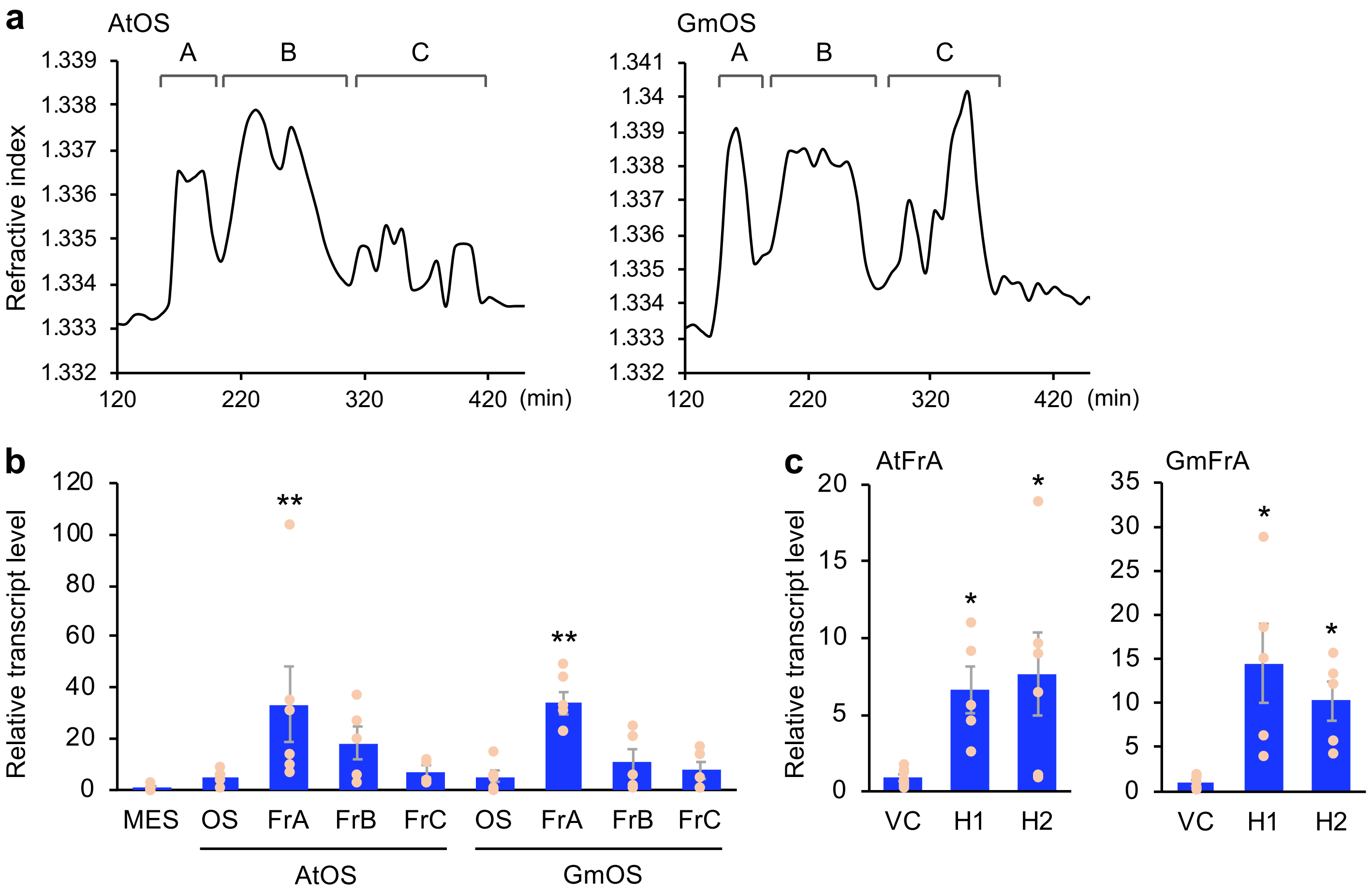

Supplementary Figure 7: Fractionation and characterization of GmHAK-mediated components in OS from *Spodoptera litura* reared on Arabidopsis or soybean (AtOS or GmOS, respectively). a Size exclusion chromatography (SEC) of Arabidopsis OS and soybean OS for components ranging in size from 100-1800 Da. **b** Transcript level of *PDF1.2* in Arabidopsis wild-type leaves at 24 h after mechanical damage with application of MES buffer, Arabidopsis OS, soybean OS or their SEC fractions (FrA, FrB or FrC). **c** Transcript level of *PDF1.2* in leaves of vector control (VC), H1, and H2 lines at 24 h after mechanical damage with application of FrA from AtOS or GmOS (AtFrA and GmFrA, respectively). All the individual data points are shown with the means and standard errors (n = 4-7). Data marked with an asterisk are significantly different from those of MES (**b**) or VC (**c**), based on an ANOVA with Holm's sequential Bonferroni post hoc test (**, $0.001 \leq P < 0.01$; *, $0.01 \leq P < 0.05$).

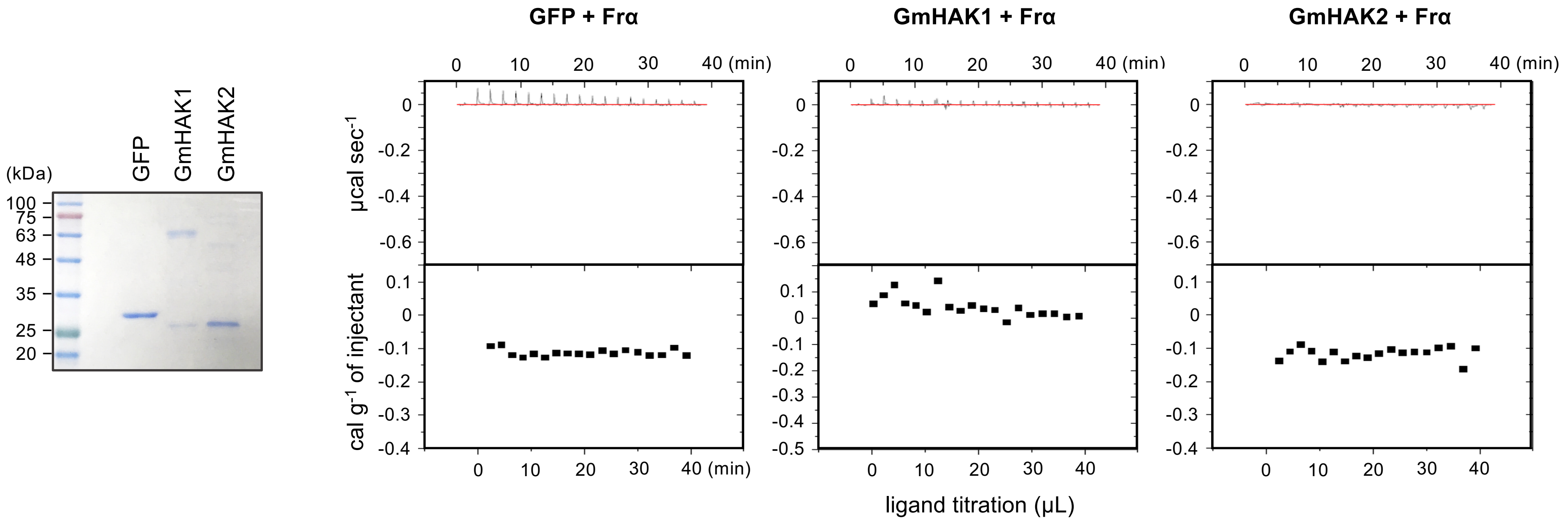

Supplementary Figure 8: Frα-binding affinity of GmHAKs. The change of heat capacity that accompanied binding of recombinant GmHAK proteins to Frα was detected using an isothermal titration calorimetry system. Upper panels and lower panels show raw data and integrated heat values, respectively. Data from Coomassie blue-stained SDS-PAGE gel of recombinant GFP and the extracellular domains of GmHAK1, and GmHAK2 proteins used are presented in the left panel.

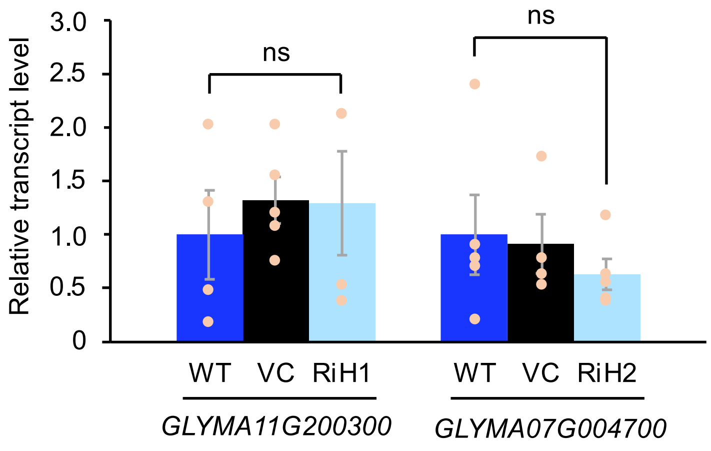

Supplementary Figure 9: GmHAK homolog expression in GmHAK RNAi lines. Transcript levels of genes for *GmHAK1* homolog (*GLYMA11G200300*) or *GmHAK2* homolog (*GLYMA07G004700*) in leaves of wild-type (WT) plants, vector control (VC) plants, and RNAi lines of GmHAK1 (RiH1) and GmHAK2 (RiH2). All the individual data points are shown with the means and standard errors (*n* = 4-5). ns, not significant based on an ANOVA.

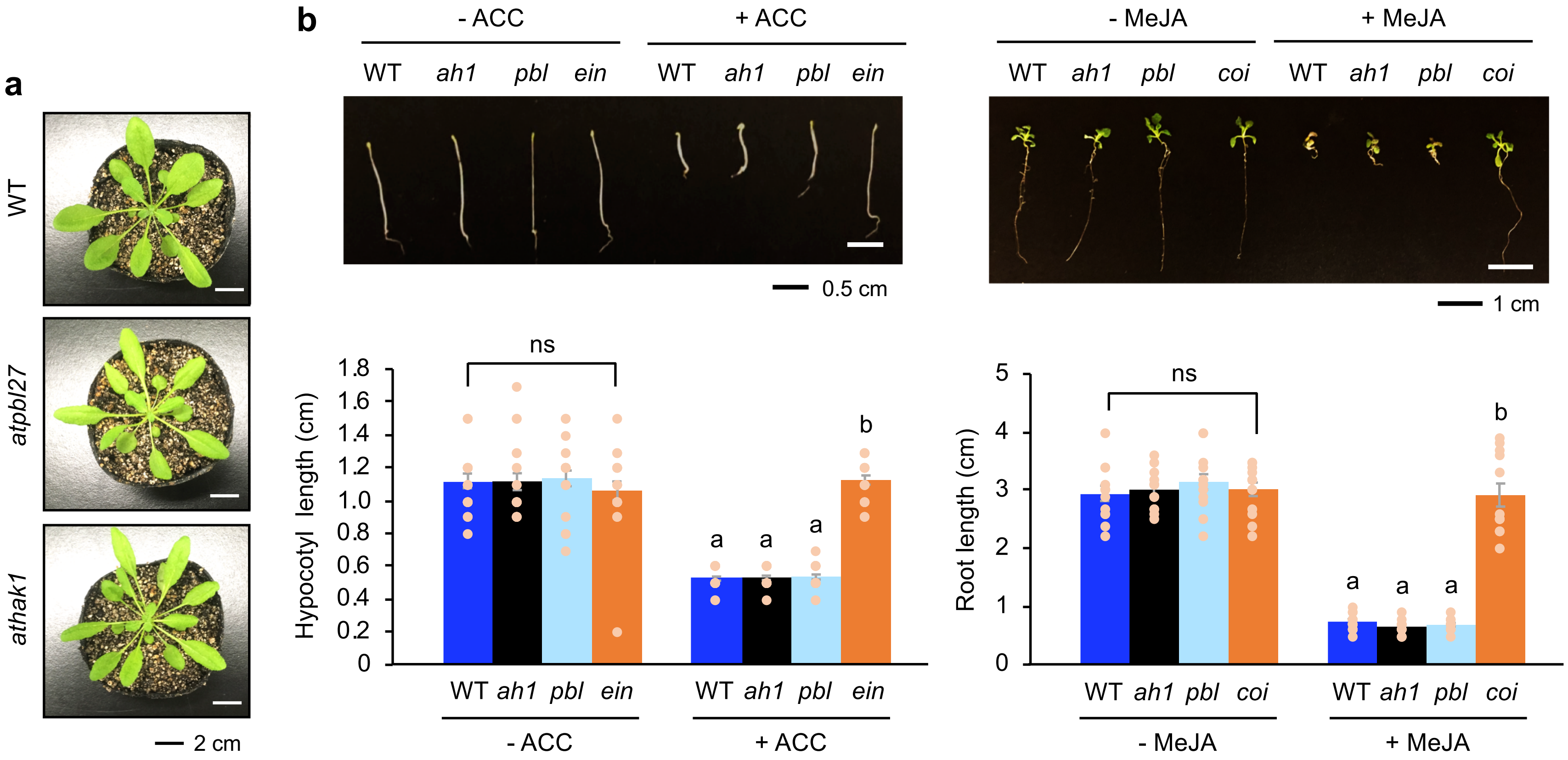

Supplementary Figure 10: The phenotypes of *athak1* and *atpbl27* mutants. a The rosette plants of wild type (WT), *athak1* and *atpbl27* grown for 4 weeks. **b** Phenotype of the sensitivity of *athak1* (*ah1*) and *atpbl27* (*pbl*) mutant seedlings to methyl jasmonate (MeJA) and an ethylene precursor 1-aminocyclopropane-1-carboxylic acid (ACC). Plant seedlings (14 days or 5 days old for MeJA and ACC, respectively) of WT, *athak1*, *atpbl27*, the coronatine-insensitive protein 1 mutant (*coi1-1* [*coi*], jasmonate insensitive mutant), and ethylene insensitive 2 (*ein2-1* [*ein*], ethylene insensitive mutant) were grown on 1/2 Murashige and Skoog medium with (+) or without (-) 50 μM MeJA or 10 μM ACC. Root and hypocotyl lengths were determined using ImageJ software (https://imagej.nih.gov/ij/). All the individual data points are shown with the means and standard errors (n = 12-14 and 20 for MeJA and ACC, respectively). The means indicated by different small letters are significantly different based on an ANOVA with post hoc Tukey's HSD ($0.001 \leq P < 0.01$).

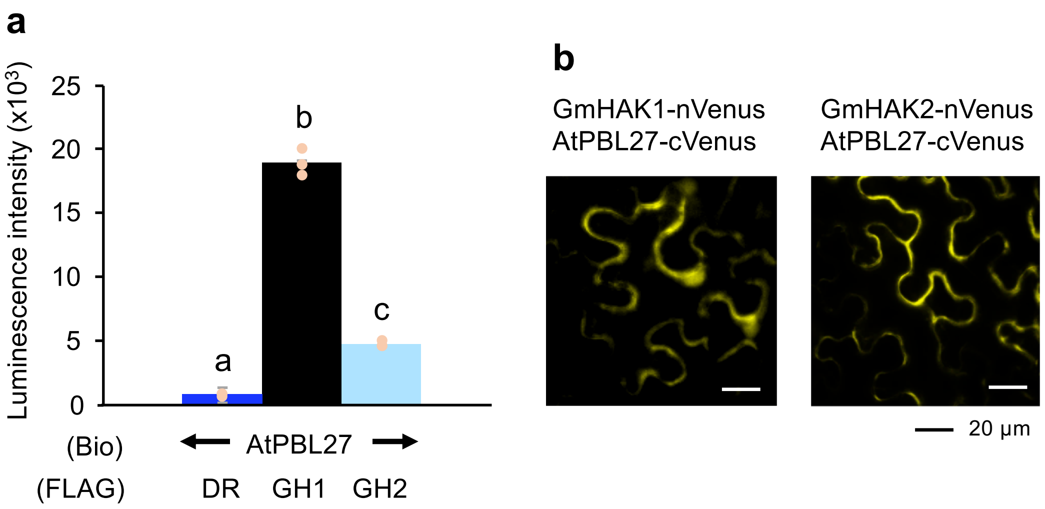

Supplementary Figure 11: Interaction of GmHAKs and AtPBL27. a Luminescence intensities based on the AlphaScreen assay to assess the interactions between biotinylated (Bio)-AtPBL27 and FLAG-conjugated proteins (FLAG) for *Escherichia coli* dihydrofolate reductase (DR) serving as control, GmHAK1 (GH1) or GmHAK2 (GH2). Proteins synthesized using the cell-free system are presented in Supplementary Fig. 12. All the individual data points are shown with the means and standard errors ($n = 3$). Means indicated by different small letters are significantly different among the respective sets of data, based on a one-way ANOVA with post-hoc Tukey's HSD ($P < 0.05$). **b** GmHAK1 or GmHAK2 fused to the N-terminal fragment of Venus (nVenus) and AtPBL27 fused to the C-terminal fragment of Venus (cVenus) was co-expressed in *Nicotiana benthamiana* leaf cells.

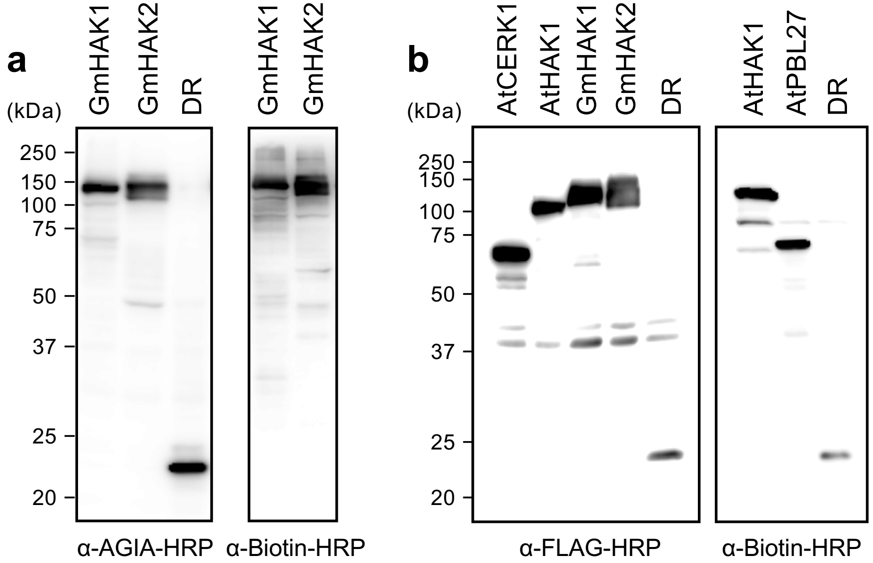

Supplementary Figure 12: Proteins used for luminescence intensities based on the AlphaScreen assay in Fig. 3a (a) and Fig. 7a (b). Data from Western blot analysis of AtCERK1, AtHAK1, AtPBL27, GmHAK1 and GmHAK2 proteins, and *Escherichia coli* dihydrofolate reductase (DR) serving as control, synthesized using the cell-free system, are presented.

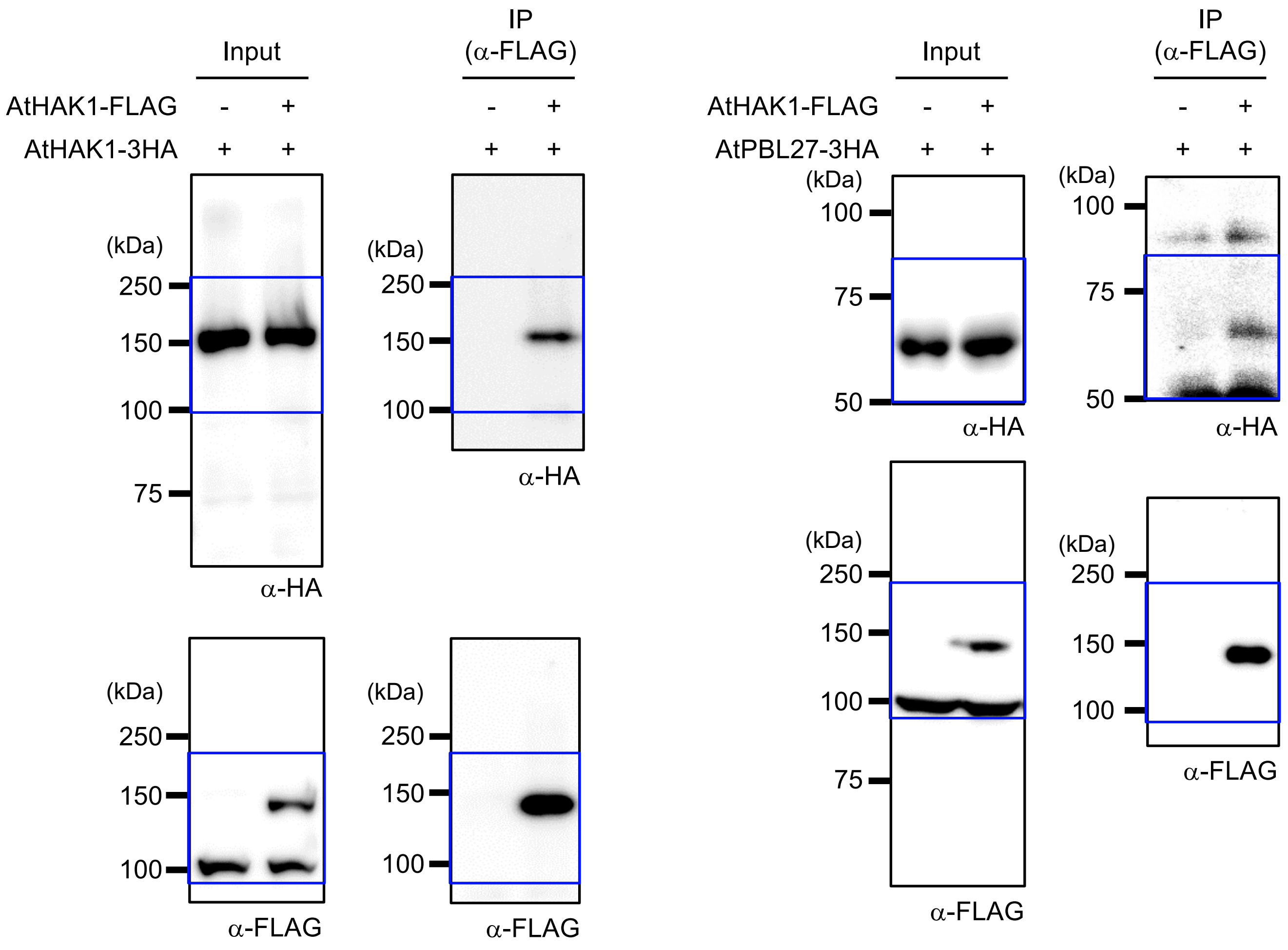

Supplementary Figure 13: Source data for Fig. 7c.
